# Metal Oxysulfides: From Bulk Compounds to Nanomaterials

**DOI:** 10.3389/fchem.2020.00179

**Published:** 2020-03-31

**Authors:** Clément Larquet, Sophie Carenco

**Affiliations:** ^1^Sorbonne Université, CNRS, Collège de France, Laboratoire de Chimie de la Matière Condensée de Paris, LCMCP, Paris, France; ^2^Sorbonne Université, CNRS, IRD, MNHN, Institut de Minéralogie, de Physique des Matériaux et de Cosmologie, IMPMC, Paris, France

**Keywords:** oxysulfide, lanthanides, sulfidation, transition metal, nanoparticles, synthesis, applications

## Abstract

This review summarizes the syntheses and applications of metal oxysulfides. Bulk compounds of rare earth and transition metals are discussed in the section Introduction. After a presentation of their main properties and applications, their structures are presented and their syntheses are discussed. The section Bulk Materials and Their Main Applications is dedicated to the growing field of nanoscaled metal oxysulfides. Synthesis and applications of lanthanide-based nanoparticles are more mature and are discussed first. Then, works on transition-metal based nanoparticles are presented and discussed. Altogether, this review highlights the opportunities offered by metal oxysulfides for application in a range of technological fields, in relation with the most advanced synthetic routes and characterization techniques.

## Introduction

### Definition

A “metal oxysulfide” is a compound composed of at least a metal, oxygen and sulfur, with negative oxidation states (e.g., –II) for both oxygen and sulfur. The generic formula for ternary oxysulfide is M_*x*_O_*y*_S_*z*_. Due to its negative oxidation state, sulfur forms no bounds with oxygen in oxysulfides, in contrast with more common metal sulfates M_*x*_(S^VI^O_4_)_*y*_ where the sulfur is +IV.

In 1951, Eastman et al. recommended the following distinction (Eastman et al., 1951): M_*x*_O_*y*_S_*z*_ compounds should be designed by the general term “oxide-sulfide” and named after the similarities of their crystalline structure with the corresponding oxide or sulfide. If the oxide-sulfide has the same crystalline structure than the oxide, it should be named “thio-oxide”; if its crystalline structure is the same than the sulfide, it should be called “oxy-sulfide” and if its structure is none of the two, it should be called “sulfoxide.”

However, in an article of 1958 published in French, Flahaut et al. questioned this nomenclature (Flahaut et al., 1958). They argued that all the Ln_2_O_2_S (Ln, lanthanide) compounds crystallize in the same structure and showed similar chemical properties. With Eastman's nomenclature, because Ln_2_O_3_ oxides crystallize in the two different structures Ce_2_O_3_ and Tl_2_O_3_, the Ln_2_O_2_S compounds would have been named “thioxyde” (French word for thio-oxide) from lanthanum to praseodymium and “sulfoxyde” (sulfoxide) for the others.

Although the terms “thio-oxide” and “oxide-sulfide” are still present in the literature, “oxysulfide” is now employed in a large majority of the works to name any combination on one or several metals to oxygen and sulfur anions.

Altogether, metal oxysulfides represent a class of compounds that is independent from both metal oxides and metal sulfides, though common properties may be punctually identified depending on the metal, the crystal structure, and the anion substitution scheme.

### Discovery and First Phases

Oxysulfides are scarce in nature and are the most often synthetic. One reason for this is the competitive formation of sulfates, which are found in numerous minerals and are more stable toward oxidation. This competition between sulfate and sulfide is also at stake when designing a synthetic route.

**Figure 1:**
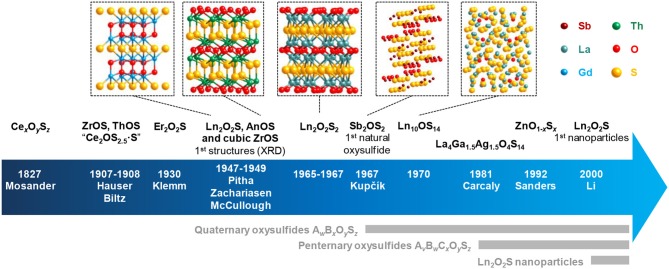
Key dates and authors of the oxysulfide research with some related structures. Ln stands for lanthanide and An for actinide. An_2_O_2_S compounds are also known since 1949 (Pu_2_O_2_S) and possess the same structure as Ln_2_O_2_S.

To the best of our knowledge, the first occurrence of an oxysulfide compound was reported in 1827 by Mosander, who was working on the sulfidation of Ce_2_O_3_ into Ce_2_S_3_ using H_2_S (Figure 1). He noticed the presence of oxygen and sulfur combined with the metal in a single product, along with the formation of cerium sulfate. Later, Sterba (1904) and Biltz (1908) also reported this observation. Biltz even proposed the formula Ce_2_S_2.5_O·S as he identified remaining sulfur as polysulfide on its final product. In 1907, Hauser prepared using H_2_S on oxides two oxysulfides of tetravalent metal, namely ZrOS and ThOS based on their composition (Hauser, 1907). Hauser indicated that the zirconium and thorium oxysulfides were pyrophoric. Without knowing it, Klemm et al. were probably the first to obtain a pure phase of Ln_2_O_2_S by heating Er(SO_4_)_3_ in H_2_S and consequently getting Er_2_O_2_S, that they only described as pale pink and resistant to other heating treatments in H_2_S (Klemm et al., 1930).

**Figure 2:**
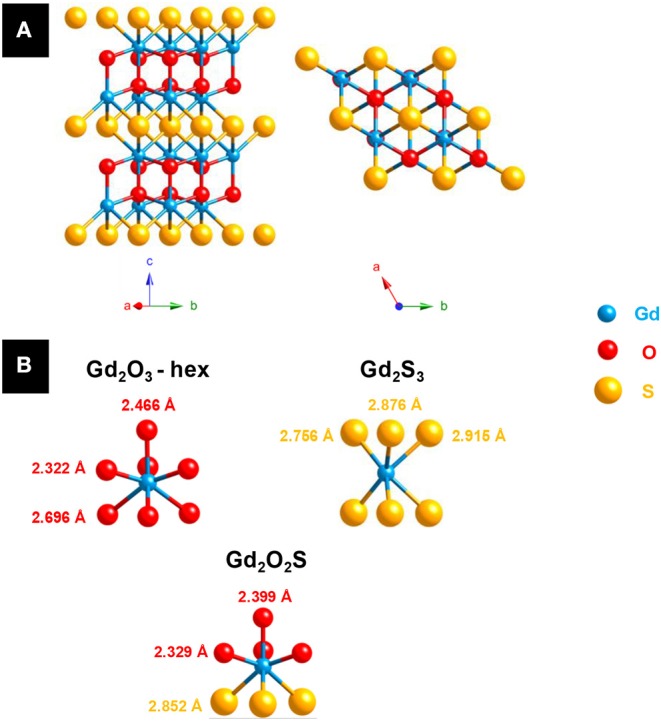
**(A)** Ln_2_O_2_S structure: a hexagonal layered structure (Ln = Gd). **(B)** Lanthanide environment in hexagonal lanthanide oxide Ln_2_O_3_ (JCPDS 02-1878), lanthanide sesquisulfide Ln_2_S_3_ (JCPDS 03-2364) and lanthanide oxysulfide Ln_2_O_2_S (JCPDS 06-8819) with the example of gadolinium.

The first crystalline oxysulfide structures were elucidated by Pitha et al. (1947) (La_2_O_2_S) and Zachariasen (1949a) (La_2_O_2_S, Ce_2_O_2_S, and Pu_2_O_2_S). The samples often contained impurities and were prepared either by reducing the corresponding sulfate Ln_2_(SO_4_)_3_ using H_2_ or by gently heating in air sesquisulfide compounds (Ln_2_S_3_). The two authors noticed that the metal was coordinated to seven atoms: four atoms of oxygen and three atoms of sulfur. The Ln_2_O_2_S structure derives from the hexagonal oxide Ln_2_O_3_ and crystallizes in the *P-3m1* space group. This lamellar structure can be described as alternating sheets of [Ln_2_O_2_]^2+^ and S^2−^ (Figure 2). Since this discovery, the entire series of lanthanide oxysulfide Ln_2_O_2_S (except promethium) was prepared (Flahaut et al., 1958).

In 1948, McCullough et al. established the structure of cubic ZrOS (McCullough et al., 1948) prepared similarly to Hauser and in 1962, Jellinek described a new tetragonal form with the same composition (Jellinek, 1962). In the 1960's and the 1970's, more M_2_O_2_S compounds were also reported. The work on radioactive elements gave the actinide oxysulfides Np_2_O_2_S (Marcon, 1967a), Am_2_O_2_S (Haire and Fahey, 1977), Cm_2_O_2_S (Haire and Fahey, 1977), Bk_2_O_2_S (Haire and Fahey, 1977), and Cf_2_O_2_S (Baybarz et al., 1974). Similarly to Ln_2_O_2_S compounds, An_2_O_2_S (An, actinide) materials crystallize in the *P-3m1* space group. On the contrary, Sc_2_O_2_S crystallizes in the hexagonal *P6*_*3*_*/mmc* space group. Its structure remains very close to Ln_2_O_2_S with a coordinence of seven for scandium atoms and a structure based on alternative layers of [Sc_2_O_2_]^2+^ and S^2−^ (Julien-Pouzol et al., 1978).

In 1949, Zachariasen described the structure of tetravalent actinide oxysulfides ThOS, UOS, and NpOS as presenting a PbFCl structure type with tetragonal symmetry in *P4/nmm* space group. The tetragonal form of ZrOS described by Jellinek is isostructural of these compounds (Zachariasen, 1949b). On the contrary, cubic HfOS (isostructural to cubic ZrOS) was first identified by Stocks et al. (1980) and prepared as a pure phase by Eisman and Steinfink (1982). The latter were also able to prepare solid solutions of zirconium and hafnium oxysulfides Zr_1−x_Hf_*x*_OS (0 ≤ *x* ≤ 1) such as Zr_0.25_Hf_0.75_OS and Zr_0.75_Hf_0.25_OS.

A few years later, Khodadad et al. and Ballestracci demonstrated the existence of several Ln_2_O_2_S_2_ compounds (Ln = La, Pr, and Nd), where the disulfide [S_2_]^2−^ anion is present (Khodadad et al., 1965; Ballestracci, 1967; Wichelhaus, 1978a). They have to be distinguished from AnOS (An, actinide) compounds in which the actinide is at the +IV oxidation state while the lanthanide in Ln_2_O_2_S_2_ remains at the +III oxidation state.

In 1967, Kupčík reported the kermesite's structure (Kupčík, 1967). The antimony-based compound Sb_2_OS_2_ is a rare crystalline natural oxysulfide mineral, which can form thanks to a partial oxidation of stibnite Sb_2_S_3_. This so-called oxydisulfide M_2_OS_2_ composition was also synthetically obtained for lanthanide compounds Ln_2_OS_2_ [Ln = Sm (Lissner and Schleid, 1992), Gd (Wontcheu and Schleid, 2003), Tb (Schleid, 1991a), Dy (Schleid, 1991b), Er (Range et al., 1990), Tm (Range et al., 1990), Yb (Range et al., 1990), Y (Schleid, 1992)]. The erbium, thulium, and ytterbium compounds were obtained at 10 kbar and 1,600°C.

A sulfur-rich phase was also discovered by trying to solve the crystalline structure of what was thought to be β-Ln_2_S_3_. It happened to be Ln_10_OS_14_ (Ln = La, Ce, Pr, Nd, Sm) that formed because of traces of water or oxygen during the reaction (Carré et al., 1970; Besançon, 1973). Besançon et al. showed that the oxygen content of Ln_10_S_14_O_*x*_S_1−x_ can be lowered down to a value close to 0.1 mol% for La, Ce, and Pr (Besançon et al., 1970, 1973). Later, Schleid et al. also reported the gadolinium compound Gd_10_OS_14_ (Schleid and Weber, 1998).

The work of Marcon with actinides led to the first description of more complex compounds, namely M^III^_2_M^IV^_2_O_4_S_3_ (Pu_4_O_4_S_3_, U_2_Pu_2_O_4_S_3_, U_2_Gd_2_O_4_S_3_, and Ce_4_O_4_S_3_), based on composition analysis (Marcon, 1967b). He also completed the work of Zachariasen by obtaining PuOS (Marcon, 1967b). In the same time, based on the work of Marcon, the compositions of cerium oxysulfides Ce_4_O_4_S_3_ (Dugué et al., 1978; Wichelhaus, 1978b) and Ce_6_O_6_S_4_ (Dugué et al., 1979) were confirmed and their structures were elucidated by X-Ray diffraction on monocrystals by Dugué et al. and Wichelhaus. Ce_4_O_4_S_3_ and Ce_6_O_6_S_4_ monocrystals were obtained by heating Ce_2_O_2_S and sulfur or CeO_2_, Ce_2_S_3_, and sulfur together. In the lanthanide series, only cerium allows both oxidation states +III and +IV. In Ce^III^_2_O_2_S, partial oxidation of cerium led to Ce^III^_2_Ce^IV^_2_O_4_S_3_ and Ce^III^_4_Ce^IV^_2_O_6_S_4_.

A decade after the discovery of Bi_2_O_2_Se (Boller, 1973), Koyama et al. published in 1984 a study about the combination of bismuth with chalcogens. They obtained the ternary oxysulfide Bi_2_O_2_S from Bi_2_O_3_ and Bi_2_S_3_ via a hydrothermal synthesis (Koyama et al., 1984). The Bi_2_O_2_S structure differs from Ln_2_O_2_S (Ln, lanthanide), as it crystallizes in the *Pnnm* space group.

**Figure 3:**
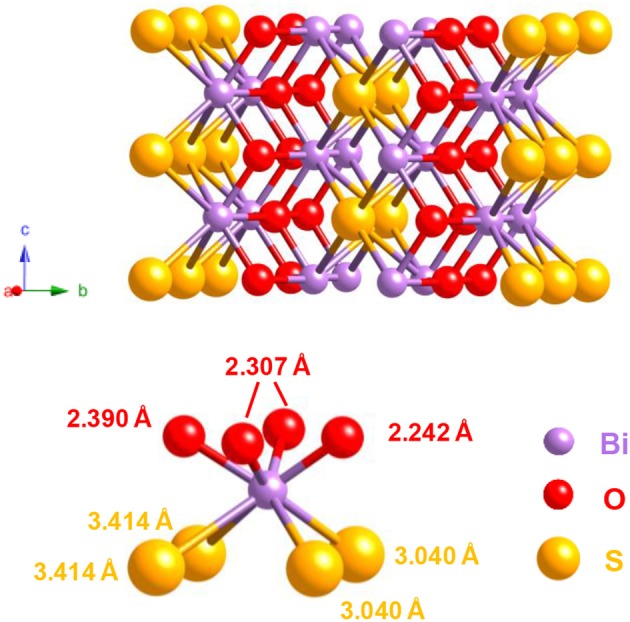
Structure of Bi_2_O_2_S (JCPDS 10-2907) and bismuth coordination in the solid.

The coordination number of the bismuth is eight: bismuth is bound to four atoms of oxygen and four atoms of sulfur (Figure 3). In comparison with Ln_2_O_2_S in which Ln forms four Ln-O and three Ln-S bonds, bismuth-oxygen bonds are in the same length range (between 2.2 and 2.5 Å) but bismuth-sulfur bonds are significantly longer (3.4 Å for Bi_2_O_2_S, <3 Å for Ln_2_O_2_S). Further works showed that bismuth can form several oxysulfides, leading to superconductive Bi_4_O_4_S_3_ (Zhang et al., 2015) (containing both sulfide and sulfate ions) and to Bi_9_O_7.5_S_6_ (Meng et al., 2015).

## Bulk Materials and Their Main Applications

### Toward More Complex Structures: Quaternary Oxysulfides and Selective Bonding

We already cited the work of Marcon who isolated actinide oxysulfides U_2_Pu_2_O_4_S_3_ and U_2_Gd_2_O_4_S_3_ (Marcon, 1967b). These structures contain U^IV^ and Ln^III^. This mixed valence allowed the formation of the An^IV^_2_Ln^III^_2_O_4_S_3_ and An^IV^_2_Ln^III^_4_O_6_S_4_ (of general formula An^IV^_2_Ln^III^_2n_O_2+2n_S_2+n_) structures by a shearing mechanism of the Ln_2_O_2_S structure when similar mixed-valent uranium-lanthanide oxysulfides were obtained (Tien et al., 1988). With Okabe et al. (1988), they also exhibited a series of U_2_La_2n−2_O_2n_S_n+1_ compounds.

Besides, in the 1980's, a considerable amount of quaternary oxysulfides containing other metals than lanthanides or actinides were synthesized. Firstly, the idea was to insert another metal in the lamellar structure of a lanthanide oxysulfide Ln_2_O_2_S. The easiest way to get a quaternary oxysulfide was to put the other metal in the layer of sulfur anions, and consequently obtain a structure composed by sheets of lanthanide oxide and metal sulfide. This compound, in which oxygen is bound only to the lanthanide and sulfur only to the additional metal, exhibits a particular order that one can call *selective bonding*. As the quaternary oxysulfides can be formed with a large variety of precursors (mainly oxides and sulfides, but also elemental sulfur, H_2_S, metals, …) and not only using lamellar preformed structures such as Ln_2_O_2_S, this selective bonding can be extended to any resulting oxysulfide in which one of the anions is preferentially bound to one of the metals and conversely. It generally led to layered compounds. On the contrary, when no such order is present in the structure (at least one metal site in the structure is bound to the two anions), the compound exhibits *unselective bonding*.

**Figure 4:**
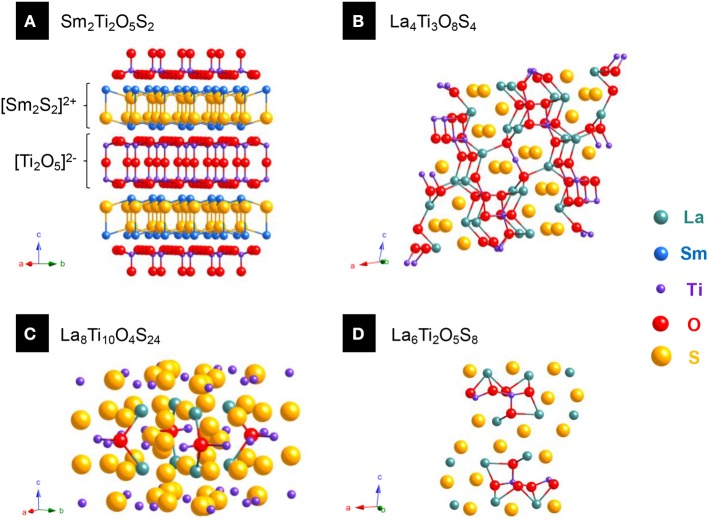
Various quarternary oxysulfide structures containing titanium. **(A)** Sm_2_Ti_2_O_5_S_2_ (JCPDS 13-1325) exhibits selective bonding as sulfur is preferentially bound to titanium and oxygen to samarium. **(B)** La_4_Ti_3_O_8_S_4_ (JCPDS 09-7018), **(C)** La_8_Ti_10_O_4_S_24_ (JCPDS 09-8085), and **(D)** La_6_Ti_2_O_5_S_8_ (JCPDS 09-7017) show different structures with unselective bonding.

To illustrate this difference, we chose to study a family of quaternary oxysulfides Ln_2_Ti_2_S_2_O_5_ (with Ti^IV^) reported in the late 1990's (Figures 4A–D). These structures turned out to be defective Ruddlesden-Popper phases which alternate [Ln_2_S_2_]^2+^ and [Ti_2_O_5_]^2−^ layers (Figure 4A). However, it is also possible to get compounds where both metals are equally bound to both oxygen and sulfur without particular arrangement (unselective bonding). It can be illustrated by the previously reported quaternary titanium oxysulfides La_4_Ti_3_O_8_S_4_ and La_6_Ti_2_S_8_O_5_ that do not show any selective bonding (Figures 4B,D; Cody and Ibers, 1995). In the 1980's, the study of the La_*w*_Ga_*x*_O_*y*_S_*z*_ compounds already started the reflexion on the selectivity of the bonds in quaternary oxysulfides (selective bonding for LaGaOS_2_-α, La_4_Ga_1.33_O_4_S_4_, and La_3_GaOS_5_; unselective bonding for LaGaOS_2_-β and La_3.33_Ga_6_O_2_S_12_, Table 1; Guittard et al., 1985).

**Table 1:** Quaternary oxysulfides M^1^_*w*_M^2^_*x*_O_*y*_S_*z*_.

**Phase**	**Space group**	**Bonding^**a**^**	**References**
An*_*w*_*An*_*x*_*O*_*y*_*S*_*z*_* and An*_*w*_*Ln*_*x*_*O*_*y*_*S*_*z*_* (An, actinide; Ln, lanthanide)			
U_0.5_Pu_0.5_OS	Tetragonal *P/nmm*	S	Marcon, 1967b
U_2_Pu_2_O_4_S_3_	Orthorhombic *Pbam*^b^	U	Marcon, 1967b
U_2_Ln_2_O_4_S_3_ (Ln = La, Ce, Pr, Nd, Sm, Gd, Tb)	Orthorhombic *Pbam*	U	Marcon, 1967b; Tien et al., 1988
U_2_Ln_4_O_6_S_4_ (Ln = La, Ce, Pr, Nd, Sm, Gd, Tb)	Orthorhombic *Pnam*	U	Tien et al., 1988
U_2_La_6_O_8_S_5_ U_2_La_8_O_10_S_6_ U_2_La_10_O_12_S_7_	Orthorhombic	U	Tien et al., 1988
U_2_LnO_2_S_3_ (Ln = Gd, Dy, Ho, Er, Tm, Yb, Lu, Y)	Tetragonal *I4/mmm*	S	Guittard et al., 1986
U_4_Lu_4_O_4_S_5_	Tetragonal *I4/mmm*	S	Jaulmes et al., 1990
Ln*_*w*_*M*_*x*_*O*_*y*_*S*_*z*_* (Ln, lanthanide or bismuth; M, 1st row transition metal)			
Ln_2_Ti_2_O_5_S_2_ (Ln = Pr, Nd, Sm)	Tetragonal *I4/mmm*	S	Boyer et al., 1999; Goga et al., 1999
La_16_Ti_5_O_17_S_17+x_(*x* = 0.75)	Tetragonal *I4/m*	U	Meignen et al., 2003
La_4_Ti_3_O_8_S_4_	Monoclinic *C2/m*	U	Cody and Ibers, 1995
La_6_Ti_2_O_5_S_8_	Monoclinic *P2_*1*_/m*	U	Cody and Ibers, 1995
La_14_Ti_8_O_4_S_33_	Monoclinic *C2/m*	U	Tranchitella et al., 1996
La_8_Ti_10_O_4_S_24_	Tetragonal *P4/mmm*	U	Cario et al., 1998
La_8.75_Ti_9.25_O_4_S_24_ La_8.50_Ti_9.50_O_4_S_24_ La_8.10_Ti_8.05_O_4_S_24_	Tetragonal *P4/mmm*	U	Tranchitella et al., 1998
La_20_Ti_11_O_6_S_44_	Orthorhombic *Pmmn*	U	Deudon et al., 1995
Ce_20_Ti_11_O_6_S_44_	Orthorhombic *Pmmn*	U	Cody et al., 1997
Nd_16_Ti_5_O_17_S_17_	Tetragonal *I4/m*	U	Boyer-Candalen et al., 2000a
Gd_6+x_Ti_4−x_S_10−y_O_6+y_	Orthorhombic *Pnma*	U	Meignen et al., 2004a
Ln_5_V_3_O_7_S_6_ (Ln = La, Ce, Pr, Nd)	Orthorhombic *Pmnm*	U	Vovan et al., 1981; Dugué et al., 1985
LaCrOS_2_	Orthorhombic *Pbnm*	U	Vovan et al., 1978; Dugué et al., 1980a
LnCrOS_2_ (Ln = Ce, Pr, Nd, Sm)	Monoclinic *B2/m*	U	Vovan et al., 1978; Dugué et al., 1980b
La_4_MnOS_6_	Hexagonal *P6_*3*_mc*	U	Ijjaali et al., 2005
Ln_2_Fe_2_O_3_S_2_ (Ln = La, Ce, Pr)	Tetragonal *I4/mmm*	U	Mayer et al., 1992; Charkin et al., 2011
LaCuOS	Tetragonal *P4/nmm*	S	Palazzi, 1981; Doussier-Brochard et al., 2010
La_5_Cu_6_O_4_S_7_	Orthorhombic *Imma*	U	Huang et al., 2000
CeCu*_*x*_*OS (*x* = 0.8; 1)	Tetragonal *P4/nmm*	S	Ueda et al., 2003; Chan et al., 2006
PrCuOS	Tetragonal *P4/nmm*	S	Lauxmann and Schleid, 2000
LnCuOS (Ln = Nd, Sm)	Tetragonal *P4/nmm*	S	Popovkin et al., 1998
BiCuOS	Tetragonal *P4/nmm*	S	Kusainova et al., 1994; Sheets et al., 2007
Ln*_*w*_*M*_*x*_*O*_*y*_*S*_*z*_* (Ln, lanthanide or bismuth; M, 2nd and 3rd row transition metal in d-block)			
La_2_Nb_3_O_8_S_2_	Orthorhombic *Pnnm*	U	Brennan and Ibers, 1992; Cario et al., 2003
La_3_MO_5_S_2_ (M = Nb, Ta)	Tetragonal *I4/mmm*	S	Cario et al., 2007
La_~10.8_Nb_5_O_20_S_10_	Orthorhombic *Immm*	U	Boyer-Candalen and Meerschaut, 2000
Ce_3_NbO_4_S_3_	Orthorhombic *Pbam*	U	Altmannshofer and Johrendt, 2008
Sm_3_NbO_4_S_3_	Orthorhombic *Pn2_*1*_a*	U	Boyer-Candalen et al., 2000b
Gd_3_NbO_4_S_3_	Orthorhombic *Pn2_*1*_a*	U	Kabbour et al., 2003
La_2_Ta_3_O_8_S_2_	Orthorhombic *Pnnm*	U	Brennan and Ibers, 1992
Sm_2_Ta_3_O_8_S_2_	Orthorhombic *Pnnm*	U	Guo et al., 1995
LaAgOS	Tetragonal *P4/nmm*	S	Palazzi et al., 1980; Palazzi and Jaulmes, 1981
CeAg*_*x*_*OS (*x* = 0.8; 1)	Tetragonal *P4/nmm*	S	Chan et al., 2006
BiAgOS	Tetragonal *P4/nmm*	S	BaQais et al., 2017
Ln*_*w*_*M*_*x*_*O*_*y*_*S*_*z*_* (Ln, lanthanide; M, 2nd and 3rd row transition metal in p-block)			
LaGaOS_2_α	Orthorhombic *P2_*1*_ab*	S	Guittard et al., 1985
LaGaOS_2_β	Orthorhombic *Pmca*	U	Jaulmes, 1978
La_3_GaS_5_O	Orthorhombic *Pnma*	S	Jaulmes et al., 1983; Guittard et al., 1985
La_3.33_Ga_6_S_12_O_2_	Tetragonal *P42_*1*_m*	U	Mazurier et al., 1982; Guittard et al., 1985
Ce_4_Ga_2_O_4_S_5_	Tetragonal *I4/mmm*	S	Jaulmes et al., 1982; Guittard et al., 1984
Ln_4_Ga_1.33_O_4_S_4_ (Ln = La, Ce)	Tetragonal *P4/mmm*	S	Guittard et al., 1985
Ln_4_Ga_2_O_4_S_5_ (Ln = Pr, Nd, Sm)	Orthorhombic *Pbca*	S	Guittard et al., 1984
LaInOS_2_	Orthorhombic	ND	Kabbour et al., 2004
La_5_In_3_O_3_S_9_	Orthorhombic *Pbcm*	S	Kabbour et al., 2004
La_10_In_6_O_6_S_17_	Orthorhombic *Immm*	S	Gastaldi et al., 1982
Ln_4_Sn_2_O_4_S_6_ (Ln = La, Ce, Pr, Nd)	Orthorhombic *Pbnm*	S	Guittard et al., 1984
LnBiOS_2_ (Ln = La, Ce, Pr, Nd, Gd, Dy)	Tetragonal *P4/nmm*	S	Céolin and Rodier, 1976; Pardo et al., 1976; Tanryverdiev et al., 1995
Ln*_*w*_*T*_*x*_*O*_*y*_*S*_*z*_* and Ca*_*w*_*T*_*x*_*O*_*y*_*S*_*z*_* (Ln, lanthanide; T, metalloid)			
Ln_4_Ge_1.5_O_4_S_5_ (Ln = La, Ce, Pr, Nd)	Orthorhombic *Pbca*	S	Guittard et al., 1984
La_4_As_2_O_4_S_5_	Tetragonal *I4/mmm*	S	Jaulmes et al., 1982
LnSbOS_2_ (Ln = La, Ce, Pr)	Not described	ND	Pardo et al., 1976
La_4_Sb_2_O_4_S_5_	X	X	Aliev and Tanryverdiev, 1997
La_6_Sb_4_O_12_S_3_	Tetragonal *I4_*1*_/amd*	U	So et al., 2004
NdSbOS_2_	Tetragonal *P4/nmm*	S	Pardo et al., 1976
CaSb_10_O_10_S_6_	Monoclinic *C2/c*	ND	Nakai et al., 1978
A*_*w*_*M*_*x*_*O*_*y*_*S*_*z*_* (A, alkaline or earth-alkaline; M, transition metal)			
K_6_Ti_6_OS_18_	Triclinic *P1*	U	Tillinski et al., 2001
Ba_6_Ti_5_OS_15_	Orthorhombic *C222_*1*_*	S	Sutorik and Kanatzidis, 1994
CaFeOS	Hexagonal *P6_*3*_mc*	S	Selivanov et al., 2004; Delacotte et al., 2015
Ca_3_Fe_4_S_3_${\text{O}}_{6}^{\text{c}}$	Tetragonal	ND	Selivanov et al., 2004
CaCoOS	Hexagonal *P6_*3*_mc*	S	Salter et al., 2016
BaCoOS	Orthorhombic *Cmcm*	U	Valldor et al., 2015; Salter et al., 2016
CaZnOS	Hexagonal *P6_*3*_mc*	S	Petrova et al., 2003; Sambrook et al., 2007
BaZnOS	Orthorhombic *Cmcm*	U	Broadley et al., 2005
SrZn_2_OS_2_	Orthorhombic *Pmn2_*1*_*	U	Tsujimoto et al., 2018
Others			
Zr_1−x_Hf*_*x*_*OS	Cubic *P2_*1*_3*	U	Eisman and Steinfink, 1982
Pb_14_Sb_30_O_5_S_54_ (scainiite)^d^	Monoclinic *C2/m*	U	Orlandi et al., 1999

a S, selective; U, unselective; ND, not described.

b Deducted from later works.

^c^Structure not solved, only based on composition.

d *Natural compound*.

### Quaternary Oxysulfides: A Large Catalog of Compositions

Using high temperatures and long reaction times, monovalent (Cu^I^, Ag^I^), trivalent (Cr^III^, Ga^III^, As^III^, Sb^III^, Bi^III^), tetravalent (Sn^IV^) and pentavalent elements (Nb^V^) were shown to be able to crystallize along with a lanthanide in various types of oxysulfide compounds (Table 1). In some cases, the second metal can also present mixed oxidation states (Ti^III, IV^, V^III, IV^).

More recently, lanthanide-free quaternary oxysulfide compounds CaMOS [M = Fe (Selivanov et al., 2004; Delacotte et al., 2015), Co (Pitha et al., 1947), Zn (McCullough et al., 1948)] and BaM'OS [with M' = Co (Pitha et al., 1947; Valldor et al., 2015), Zn (Broadley et al., 2005)] were synthesized and characterized. This shows the growing interest in obtaining metal oxysulfides without rare earth (which are strategic resources) in order to explore their magnetic and catalytic properties.

In this table are not referenced the quaternary phases reported by Umarji et al. in 1980: M_2_Mo_6_S_6_O_2_ (M = Co, Ni, Cu) and PbMo_6_S_6_O_2_ (Umarji et al., 1980). A few years after this publication, Selwyn et al. tried to obtain the copper-based phase and demonstrated that Umarji et al. reached only a mixture of the Chevrel phase Cu_2.7_Mo_6_S_8_, Mo, and MoO_2_ (Selwyn et al., 1987). Then Selwyn et al. also concluded that obtaining the ternary Mo_6_S_6_O_2_ oxysulfide from the claimed M_2_Mo_6_S_6_O_2_ was impossible.

### Quinary Oxysulfides

Quinary oxysulfides also exist, but are not exhaustively listed in this review. Most of them are layered compounds with selective interactions and contain earth-alkaline atoms, as evidenced by Teske in 1985 with CaLaGa_3_OS_6_, SrLaGa_3_OS_6_, La_2_ZnGa_2_OS_6_, and Sr_2_ZnGe_2_OS_6_ (Teske, 1985). A similar Sr_2_MnGe_2_OS_6_ phase was synthesized and studied recently (Endo et al., 2017). Doped phosphors CaLaGa_3_OS_6_ (Yu et al., 2008, 2012; Zhang et al., 2010, 2011, 2012) and SrLaGa_3_OS_6_ (Zhang et al., 2005a, 2016; Yu et al., 2011, 2012) were extensively studied by Zhang, Yu, and Zhang since 2005. Zhu, Hor, and Otzschi also detailed different quinary oxysulfide families: (i) the Sr_2_Cu_2_MO_2_S_2_ [M = Mn (Zhu and Hor, 1997a), Co (Zhu et al., 1997; Smura et al., 2011), Zn (Zhu and Hor, 1997a), Ni (Otzschi et al., 1999)] and Ba_2_Cu_2_CoO_2_S_2_ (Zhu et al., 1997; Smura et al., 2011) family that displays an unusual square planar MO_2_ layer and the two perovskite-based families (ii) Sr_3_Cu_2_M_2_O_5_S_2_ [M = Sc (Otzschi et al., 1999), Fe (Zhu and Hor, 1997b)] and (iii) Sr_2_CuMO_3_S [M = Sc (Ogino et al., 2012), Cr (Zhu and Hor, 1997b), Fe (Zhu and Hor, 1997b), Ga (Zhu and Hor, 1997c), In (Zhu and Hor, 1997b)] with the work of Ogino on scandium. Later, Blandy transformed Sr_2_Cu_2_MnO_2_S_2_ in Sr_2_Cu_1.5_MnO_2_S_2_ by oxidative deintercalation of copper to obtain a mixed-valent perovskite (Blandy et al., 2015).

The study of the quasi-binary system La_2_O_2_S-AgGaS_2_ (La_2_O_2_S – 0.75 Ga_2_S_3_ – 0.75 Ag_2_S) by Carcaly et al. (1981) led to the formation of La_4_Ag_1.5_Ga_1.5_O_4_S_5_ in which silver and gallium are randomly distributed in the same sites. Along with La_3_MO_5_S_2_ (M = Nb, Ta; Table 1), Cario et al. reported bilanthanide lamellar La_2_YMO_5_S_2_ phases very close to the Ln_2_Ti_2_O_5_S_2_ structure (Eisman and Steinfink, 1982). The works of Tranchitella on La/Ti quaternary oxysulfide (Table 1) led him to the quinary compound Sr_5.8_La_4.4_Ti_7.8_S_24_O_4_ with the same [(Ti_4_S_2_O_4_)(TiS_6_)_4/2_]^12−^ layer than La_14_Ti_8_S_33_O_4_ (Tranchitella et al., 1996). La_5_Ti_2_MS_5_O_7_ (M = Cu, Ag), an alkaline-free structure with perovskite layers was also evidenced by Meignen et al. (2004b) and studied for its photocatalytic properties for water reduction and oxidation (Suzuki et al., 2012). Meignen et al. (2005) also prepared La_5_Ti_~3.25_Zr_~0.25_S_5_O_9.25_ with mixed Ti/Zr sites. In 2003, Rutt et al. obtained KY_2_Ti_2_O_5_S_2_ by topotactic potassium intercalation of potassium in Y_2_Ti_2_O_5_S_2_ (Rutt et al., 2003). As a perspective, in 2015, Yee et al. designed by DFT modeling a new high-temperature superconductor Ca_2_HgCuO_2_S_2_ whose superconducting transition temperature should be close to mercury cuprates' ones (Yee et al., 2015).

### Transition Metal Oxysulfides

For a long time, ternary oxysulfides M_*x*_O_*y*_S_*z*_ were limited to lanthanides, actinides, and bismuth. Despite the presence of numerous metals in quaternary oxysulfides, the transition metals did not give any crystalline ternary oxysulfide (except ZrOS and HfOS) until the synthesis of ZnO_1−x_S_*x*_ in the 1990's. This phase is the most often found as crystalline thin films. It is also the case for titanium, tungsten and molybdenum oxysulfides except that they are amorphous.

The first-raw transition metals ternary oxysulfides represent a challenge, because the coordination of the metal commonly does not exceed six, and consequently cannot bear the M_2_O_2_S structure of Ln_2_O_2_S (Ln, lanthanide) where the lanthanide coordination is seven or the Bi_2_O_2_S structure where the coordination of bismuth is eight. Alternative crystal structures may be obtained in the case of first-raw transition metals.

#### Crystalline Transition Metal Oxysulfides

##### Copper oxysulfide Cu_*2*_O_*1−*x**_S_*x*_

In 2013, Meyer et al. reported the synthesis of ternary compounds Cu_2_O_1−x_S_*x*_ with various compositions (Meyer et al., 2013). These were obtained using radio-frequency magnetron sputtering (RFS) with a copper target and a flow of O_2_ and H_2_S with various gas ratios. The authors showed that for *x* > 0.39, the compounds did not crystallize in the cubic structure of Cu_2_O and became amorphous. The lattice constant of cubic Cu_2_O_1−x_S_*x*_ evolved with the composition toward bigger values because of sulfur insertion. The variation was linear only up to *x* = 0.13 and did not follow the Vegard's law. Unfortunately, direct information about the sulfur oxidation state is missing: the oxysulfide nature of the compound remains unsubstantiated.

##### Zinc oxysulfide ZnO_*1*−*x*_S_*x*_

Despite their electronegativity and size differences, sulfur atoms are able to replace the oxygen atoms of the wurtzite structure which progressively turns into the ZnS blende structure. It evidences another challenge of metal oxysulfide identification: they could be isostructural of metal sulfides or metal oxides.

Zinc oxysulfide was first reported as thin films grown by atomic layer deposition (ALD) in 1992 by Sanders et al. The oxygen and water traces in the gases were responsible for the oxygen in the resulting film. Since 2010, extensive characterization of ZnO_1−x_S_*x*_ thin films were reported, not only involving ALD (Bakke et al., 2012) but also pulsed-laser deposition (Deulkar et al., 2010), chemical spray pyrolysis (Polat et al., 2011a,b, 2012; Thankalekshmi and Rastogi, 2012) or thioacetate-capped ZnO nanocrystals (Lee and Jeong, 2014). Because of the active research on bandgap engineering, zinc oxysulfide was envisaged as buffer layer in solar cells (Platzer-Björkman et al., 2006; Sinsermsuksakul et al., 2013). X-Ray photoemission spectroscopy (XPS) showed that the sulfur in these films is reduced and thus in agreement with the announced oxysulfide nature (Thankalekshmi and Rastogi, 2012; Lee and Jeong, 2014).

##### Molybdenum oxysulfides *Mo*_*x*_*O*_*y*_*S*_*z*_

In 1986, Inoue et al. crystallized two Mo_*x*_O_*y*_S_*z*_ compounds while studying the MoS_2_:MoS_3_ system (Inoue et al., 1986). The deep-bluish crystal of MoO_2.74_S_0.12_ (otherwise written as Mo_4_O_10.96_S_0.48_) was isostructural to γ-Mo_4_O_11_ and exhibited charge density wave instabilities similar to these of quasi-2D materials. The similar properties of MoO_2.74_S_0.12_ and γ-Mo_4_O_11_ supported the hypothesis of a true oxysulfide compound. Also, reddish crystals of MoO_1.88_S_0.15_ were obtained and presented structural and electronic similarities with monoclinic MoO_2_.

The decomposition of molybdenum oxodithiocarbamate as a single source precursor also enabled the formation of crystalline thin films (Olofinjana et al., 2010). Rutherford backscattering spectroscopy (RBS) indicated a pure phase. Unfortunately, the final product shared the XRD patterns of Mo_8_O_23_, Mo_9_O_26_, and Mo_2_S_3_ but the structure was not fully solved.

#### Amorphous Titanium, Tungsten, and Molybdenum Oxysulfides

In this section are referenced the oxysulfides of three elements: titanium, tungsten, and molybdenum. In the 1990's, thin films of these oxysulfides were obtained and studied for their electrochemical properties.

In 1993, Tchangbedji et al. announced the formation of a hydrated amorphous phase of vanadium oxysulfide by reacting Na_2_S·9H_2_O and VOCl_2_ (Tchangbedji et al., 1993). The first described formula for this compound was V_2_O_4_S·2H_2_O, but was adjusted to V_2_O_3_S·3H_2_O in latter studies (electron paramagnetic resonance and XANES at V K-edge demonstrated the presence of V^IV^ species; Tchangbédji et al., 1994; Ouvrard et al., 1995). Water was believed to stabilize the compounds, as its evaporation was accompanied by the loss of the sulfur in the structure. Unfortunately, the authors did not provide enough convincing arguments to justify the oxysulfide nature and the purity of their phase without ambiguity. In particular, the absence of the IR and XANES at S K-edge spectra, which are discussed in the articles, is detrimental. Because of this lack of information, we did not focus on this phase.

##### Titanium

Titanium oxysulfides were obtained under the form of thin films to serve as positive electrode material for solid state batteries. Reported for the first time in 1989 by Meunier et al. (1989, 1991) they were extensively characterized in the same group by X-ray photoemission spectroscopy (XPS) that was shown well-adapted for thin films characterization (Levasseur et al., 1999).

Titanium oxysulfides (TiO_*y*_S_*z*_) of various compositions were obtained using RFS of hydrolyzed TiS_2_ targets. The composition can be adjusted via the partial pressure of oxygen during the sputtering process. XPS showed that titanium oxysulfides thin films contain three titanium species (Ti^IV^ as in TiO_2_, Ti^IV^ as in TiS_2_ and Ti in mixed environment) and three sulfur species (S^−II^ of S^2−^ anions, S^−I^ in disulfide S_2_^2−^ ions and undefined S_n_^2−^ ions). For high oxygen contents (TiOS for instance), S^VI^ species of sulfate ions attributed to surface species were also observed, although in a lesser extent due to mechanical erosion (Gonbeau et al., 1991; Dupin et al., 2001; Martinez et al., 2004; Lindic et al., 2005a) Besides, the presence of ordered domains, observed by TEM and XRD, revealed the existence of TiS_2_ nanocrystals in the amorphous materials (Lindic et al., 2005b). Lithiated titanium oxysulfides thin films were recently obtained with RFS using LiTiS_2_ targets (Dubois et al., 2017). Their characterization show similar properties than TiO_*y*_S_*z*_. Their capacities of around 85 μAh.cm^−2^.μm^−1^ made them usable in a Li-ion cell.

Aside these thin films, “sulfur-doped TiO_2_” can be obtained by reacting TiO_2_ with thiourea or hexamethyldisilathiane, for instance. However, in this case, the products should not be named “oxysulfides,” because they only contain oxidized sulfur under the form of S^IV^ and S^VI^ species (Yang et al., 2012; Ramacharyulu et al., 2014; Smith et al., 2016).

##### Tungsten

Similarly to titanium oxysulfides, amorphous tungsten oxysulfides thin films with adjustable composition were obtained by RFS on WS_2_ targets and mainly characterized by XPS (Martin et al., 1999). Along with the three species of sulfur described in the titanium section, three different species of tungsten (W^VI^ as in WO_3_, W^IV^ as in WS_2_, and W^V^ in a mixed environment of O^2−^, S^2−^, and S_2_^2−^) were observed (Dupin et al., 2001; Martinez et al., 2004). TEM and XRD showed the presence of nano-crystallites of WS_2_, but the polymorphs 3R-WS_2_ and 2H-WS_2_ could not be distinguished (Martin-Litas et al., 2002). The incorporation of lithium in these thin films and their electrochemical properties were studied (Martin et al., 1999; Martin-Litas et al., 2001). It revealed that 1.1 lithium atoms per formula can be incorporated, providing a capacity of 75 μA.cm^−2^. XPS also demonstrated that the tungsten ions are reduced to W^(0)^ and that sulfide ions participated to the redox process with irreversible behaviors (Martin-Litas et al., 2003).

##### Molybdenum

Abraham, Pasquariello et al. synthesized various MoO_*y*_S_*z*_ amorphous compounds from the thermal decomposition of ammonium dithiomolybdate (NH_4_)_2_MoO_2_S_2_ (Abraham et al., 1989; Pasquariello et al., 1990). This precursor was obtained by bubbling H_2_S on ammonium paramolybdate [(NH_4_)_6_Mo_7_O_24_·4H_2_O] in an ammonia solution. Depending on the thermal treatment (temperature, number of steps), significant amounts of hydrogen and/or nitrogen could be found in the solids. Reacting a mixture of [(NH_4_)_6_Mo_7_O_24_·4H_2_O] and (NH_4_)_2_MoS_4_ also led to a solid precursor whose thermal decomposition yielded MoO_*y*_S_*z*_. Based on the electrochemical properties of these amorphous compounds, the authors suggested different structures for them, with different O/S ratios and involving both S_2_^2−^ and S^2−^ anions (Abraham and Pasquariello, 1993). Infrared spectroscopy and XPS supported the presence of Mo–O and Mo–S bonds in the solid, but Mo–Mo bonding could not be evidenced.

The solution obtained by reflux of (NH_4_)_2_Mo_2_S_12_ in acetone dispersed in different aqueous electrolyte solutions led to original morphologies of amorphous molybdenum oxysulfides (water/acetone = 1/10 *v*/*v*; Afanasiev and Bezverkhy, 2003). For instance, tubular morphologies (with the following electrolyte: 10% KCl, 10% NH_4_SCN), hollow spheres [with 10% (NH_2_OH)H_2_SO_4_] and fractal sponge-like solids (with 20% NH_4_SCN) were obtained. A solid was collected by evaporation of the solvent. EXAFS at Mo K-edge spectra showed one oxygen atom and four sulfur atoms in the first coordination shell of molybdenum atoms. XPS supported the hypothesis of mainly reduced sulfur, even if a broad peak in the region 167–171 eV indicated oxidized species. In the same group, Genuit et al. (2005) performed the condensation in acidic medium of MoO_2_S_2_^2−^ to amorphous MoOS_2_. The addition of HCl in a (NH_4_)_2_MoO_2_S_2_ aqueous solution led to MoOS_2_.

Similarly to titanium and tungsten, RFS gave amorphous thin films of molybdenum oxysulfides (Schmidt et al., 1994, 1995a). The target was a pellet of MoS_2_. Pure oxygen was flowed into the chamber to get oxygen-rich oxysulfides (MoO_~1.3_S_~1.9_), but the traces of oxygen in the glovebox were originally sufficient to get MoO_*y*_S_*z*_ thin films. For oxysulfides with a low content of oxygen (MoO_~0.5_S_~2.0_), TEM showed ordered domains that are isostrucural of MoS_2_, based on electronic diffraction. XRD evidenced both MoS_2_ and MoO_2_ phases when the films were annealed under inert atmosphere. As shown by Buck for contaminated MoS_2_ films, substitution of sulfur by oxygen atoms is likely to explain the changes in lattice parameters observed in the MoS_2_-like phase (Buck, 1991). Later, XPS analysis provided clues about the oxidation states of molybdenum and sulfur in MoO_*y*_S_*z*_ films which strongly varied with the film composition (Levasseur et al., 1995; Schmidt et al., 1995b; Dupin et al., 2001). For *y* <0.6 and *z* > 2 (oxygen-poor oxysulfides), Mo^IV^ cations and S^−II^ (as in MoS_2_) were dominant. For *y* > 3 and *z* <1 (oxygen-rich oxysulfides), only Mo^VI^ in octahedral sites (as in MoO_3_) was observed. For 0.6 < *y* <3 and 1 < *z* <2, Mo^V^ was observed in addition to Mo^IV^ and Mo^VI^ and was likely surrounded by O^−II^ (O^2−^) and S^−I^ (S_2_^2−^) species. Moreover, in MoO_0.6_S_1.9_ thin films, extended X-ray absorption fine structure (EXAFS) at molybdenum K-edge also showed the presence of oxygen atoms in the coordination sphere of molybdenum atoms (Schmidt et al., 1995b).

During the same decade, useful XPS and IR references for molybdenum oxysulfides were established by Muijsers et al. (1995) and Weber et al. (1996) in the study of MoO_3_ films sulfidation. The formation of oxysulfide intermediate species with their corresponding probable structures was detailed.

### Applications of Bulk and Thin Film Metal Oxysulfides

In the 1980's, potential applications for doped Ln_2_O_2_S materials were identified and led to their use as lamps, lasers, scintillators, screens, etc. For example, they can be found in X-ray detectors used for tomography or medical imaging. They have also been used for oxygen storage. Often, Y_2_O_2_S, La_2_O_2_S, or Gd_2_O_2_S are used as the lattice and doped with one or several lanthanide ions to obtain the desirable luminescence features. The oxysulfide was compared with the corresponding oxide Sm_2_Ti_2_O_7_ that has a higher bandgap. Also, electrochemical properties of transition metal oxysulfides were investigated for their use in lithium-ion batteries. A brief summary is given below on these applications.

#### Screens

Doped oxysulfides were primary employed in cathode ray tubes (CRTs) of television screens and later in computer monitors. In 1968, Royce patented a “family of new cathodoluminescent phosphors” by describing the potential use of doped Y_2_O_2_S and Gd_2_O_2_S (Royce, 1968). Lutetium and lanthanum were also envisaged as efficient matrixes for the doping ions (mainly Sm^III^ or Eu^III^).

Three classes of phosphors, respectively, associated to red, blue, and green, are necessary for a proper screen to emit the colors of the visible spectrum. Thanks to their good luminescence properties, lanthanide doping ions equip the main phosphors used for industrial applications (Jüstel et al., 1998). Red color is provided by Eu^III^: Y_2_O_3_:Eu, Y_2_O_2_S:Eu, YVO_4_:Eu, Y_2_(WO_4_)_3_:Eu; blue emission is enabled by Eu^II^ in compounds such as: Sr_5_(PO_4_)_3_Cl:Eu, BaMgAl_11_O_7_:Eu, Sr_2_Al_6_O_11_:Eu; and green is emitted thanks to Tb^III^: CeMgAl_11_O_19_:Tb, (Ce,Gd)MgB_5_O_10_:Tb, (La,Ce)PO_4_:Tb, Y_2_SiO_5_:Tb, Y_3_Al_5_O_12_:Tb (Ronda et al., 1998). However, for CRTs, blue and green are preferentially obtained with ZnS:Ag and ZnS:(Cu,Au), respectively.

In current computer monitors, the amount of europium-doped yttrium oxysulfide Y_2_O_2_S:Eu (0.73% w/w for Eu, 13.4% w/w for Y) used for red emission has become large enough to implement and develop the rare-earths recovery (Resende and Morais, 2015).

#### Laser: Emission and Absorption

##### Stimulated emission in lanthanum oxysulfide

The first study on metal oxysulfides as laser-emitting material was reported in the earliest years of the design of laser devices. “Laser” stands for light amplification by stimulated emission of radiation and is a general term for a device that emits light through a process of optical amplification based on the stimulated emission of electromagnetic radiation. It is characterized and differs from other light sources by the spatial and temporal coherences of the resulting light. Thus, there are countless applications of the laser devices. Two kinds of applications can be distinguished: information transfer (fiber-optic communication, length measurements, fingerprint detection, barcode scanner, thermometers, laser pointers, weapon guidance…) and power transfer (cutting, welding, superficial fusion, marking materials…).

In 1971, while the most famous laser crystal, namely YAG:Nd (neodymium-doped yttrium aluminum garnet), had already been extensively studied, Alves et al. (1971) carried out the first experiments dealing with an oxysulfide-based laser. They grew and studied millimetric La_2_O_2_S:Nd crystals with many defects, but also estimated the properties of crystals with less imperfections. Similarly to YAG:Nd, the stimulated emission takes place between the ^4^F_3/2_ and ^4^I_11/2_ energy levels of the Nd^III^ ion in La_2_O_2_S:Nd with an emission wavelength of 1,075 nm (9,300 cm^−1^), while YAG:Nd emits at 1,064 nm (9,400 cm^−1^).

In 1990, Markushev et al. (1990) presented preliminary results on the stimulation emission kinetics at the temperature of liquid nitrogen for 1 mol% of neodymium. In 2012, the stimulated emission properties of La_2_O_2_S:Nd were studied with oxysulfide powders (Iparraguirre et al., 2012). In particular, Iparraguirre et al. (2012) estimated and experimentally investigated the influence of the doping ion concentration and pumping wavelengths on the different laser properties.

##### Laser absorption of samarium oxysulfide

The counterpart of laser emission is laser absorption. Because of the coherence of the emitted light, laser devices can be harmful for human skin or eyes. Protecting glasses or clothes are then required for safety issues. Absorption materials must display a low reflectivity and a good thermal stability because of local heating induced by the laser beam.

The research on absorption devices deals with materials that can absorb the 1,064 nm radiation of the widespread YAG:Nd^3+^ laser. In particular, samarium-based compounds were found to be efficient absorption materials because of electronic transitions between the ground state ^6^H_5/2_ to the ^6^F_9/2_ excited state.

Undoped Sm_2_O_2_S was found to absorb a large proportion of the 1,064 nm laser radiation with a reflectivity of around 0.74% and it is stable up to 2,000°C (Zhu et al., 2016). In comparison, SmBO_3_ presents a reflectivity of 0.6% but endures a phase transition at 1,200°C (He et al., 2009). Doping with erbium or thulium may also be an efficient way to slightly enhance the absorption properties of Sm_2_O_2_S (Sun et al., 2017).

#### Scintillators

A scintillator is a material that emits light when it is excited by an ionizing radiation (X-rays or gamma rays for example). Scintillators are mainly used in the field of medical imaging. Their role is to lower the dose of X-rays endured by a patient during an analysis. To enable a good absorption of the X-ray beam, the requirements for a good scintillator phosphor is the presence of heavy atoms (cadmium, bismuth, lanthanides, tungsten for instance), a high material density (≥ 4 g.cm^−3^) and a high stability regarding the radiations. The photon must be converted into photons in the visible range (500-800 nm) with a good efficiency, a fast decay and a short afterglow. Moreover, mechanical strength, absence of toxicity, and chemical stability are desired features (Rossner and Grabmaier, 1991).

Thus, a scintillator is generally composed by a dense ceramic and converts X-rays in visible light. It is connected to photodiodes that convert the visible photons in electrons that form an image on a layer of amorphous silica. Considering their efficient absorption of X-rays, Y_2_O_2_S:Tb, La_2_O_2_S:Tb, Gd_2_O_2_S:Tb were considered to replace CaWO_4_, which was commonly used as scintillator (Brixner, 1987). Gd_2_O_2_S:Tb was finally chosen for its higher density and better absorption properties in comparison to the other lanthanides (Brixner, 1987). Later, Gd_2_O_2_S:Pr was shown to be an efficient scintillator by Rossner et al. They demonstrated that the main differences between the Pr^III^ and the Tb^III^ doping lie in the incident beam conversion efficiency (for a 40-80 keV X-ray beam, 8.5% for Gd_2_O_2_S:Pr, Ce, F, and 15% for Gd_2_O_2_S:Tb) and the luminescence lifetime of the doping ion (~3 μs for Gd_2_O_2_S:Pr, Ce, F; 600 μs for Gd_2_O_2_S:Tb; Rossner and Grabmaier, 1991). Pr^III^ shows a very rapid decay, cerium decreases the trap states and fluorine causes an important decrease of the afterglow. Gd_2_O_2_S:Eu was also studied. Its absorption and luminescence properties were competitive enough and it enables the emission of red photons (instead of green photons for Pr and Tb) which can be useful for compatibility issues with digital imaging systems (Michail et al., 2010).

Nowadays, gadolinium oxysulfides are used as scintillators for Single-Photon Emission Computed Tomography (SPECT), X-ray Computed Tomography (CT), and Positron Emitting Tomography (PET).

#### Lithium-ion Batteries

Lithium intercalation and electrochemical properties of bulk metal oxysulfides were discussed because of possible oxido-reduction reactions with transition metals such as titanium or molybdenum. We already mentioned that titanium (Meunier et al., 1989; Lindic et al., 2005a; Dubois et al., 2017) or tungsten (Martin et al., 1999; Martin-Litas et al., 2001, 2003) thin films were studied as cathodes in solid-state lithium-ion batteries. As cathodes, molybdenum oxysulfide thin films were also developed (Abraham et al., 1989; Pasquariello et al., 1990; Gonbeau et al., 1991; Abraham and Pasquariello, 1993; Levasseur et al., 1995; Schmidt et al., 1995a; Yufit et al., 2003; Golodnitsky et al., 2006a,b) More recently, a TiO_2_@MoO_*y*_S_*z*_ composite was investigated as anode material (Qiao et al., 2013). The external layer of molybdenum oxysulfide was supposed to enhance the conductivity of the hybrid material.

### Conclusion

At the end of this section, we wanted to underline several crucial points:

- Oxysulfide materials are mainly reached by chemical synthesis and much fewer compositions were obtained compared to monochalcogenide compounds.
- The lack of oxysulfide compositions is mainly based on the strong differences between oxygen and sulfur. Metals tend to preferentially bind to one compared to the other.
- Transition metals oxysulfides are particularly rare and their crystalline phases even more.

**Figure 5:**
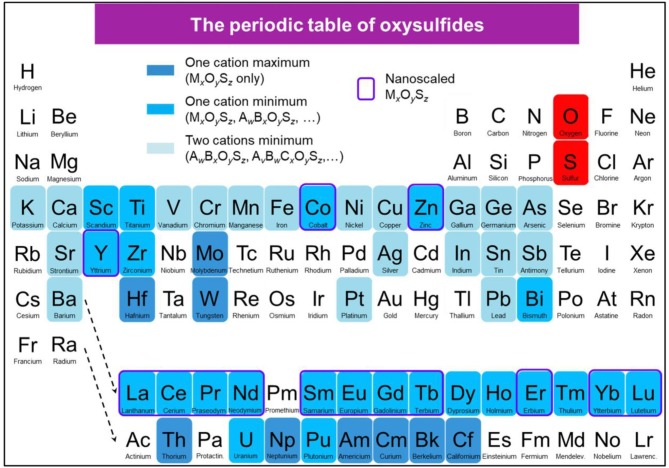
Periodic table showing the reported oxysulfide compounds. In blue are indicated the elements that can be found in synthetic or natural oxysulfides. Blue shades indicate the compositions (ternary, quaternary, and more) that can be achieved for each element. Surrounded in violet are the elements for which M_*x*_O_*y*_S_*z*_ nanoparticles were reported.

In this context, a periodic table showing the reported oxysulfide compounds is presented in Figure 5.

## Nanoscaled Ternary Lanthanide Oxysulfides Ln_2_O_2_S

### Introduction

**Figure 6:**
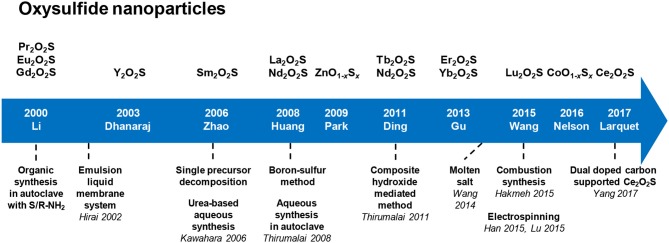
Key dates, authors, and techniques of the oxysulfide nanoparticles research.

Following the hype for nanotechnology in the twenty-first century, researchers have recently worked on producing metal oxysulfide materials as nanoparticles (Figure 6). This trend is justified by the applications that could emerge from nanomaterials, especially in the domain of biology and medicine. In particular, nano-scale objects can cross biologic barriers and be metabolized by living beings. Also, because of their wide range of morphologies, compositions, and grafting, the nanoparticles can reach targeted zones using specific interactions to provide local information, deliver drugs at precise places or stimulate organs and tissues with an internal or external stimulus.

Lanthanide oxysulfide nanomaterials present many advantages for imaging in biological medium. They have a good chemical and thermal stability. Their size and shape is highly tunable from very small crystals around 5 nm to micrometer spheres, rods, belts, tubes and so on. Moreover, the Ln_2_O_2_S crystalline phase bears many lanthanide/transition metal or lanthanide/lanthanide substitutions, which guarantees a generous variety of luminescent properties.

#### Examples of Oxysulfide Nanoparticles' Applications

##### Upconversion

In the fields of therapy and *in vivo* imaging, using direct light composed of high energy photons, typically X-rays or gamma rays, leads to potential harmful effects for the patient. Organic dyes, radioisotopes and quantum dots are currently used in order to perform bioimaging. However, toxicity of radioactive isotopes, and quantum dots is problematic. Also, organic fluorophores and quantum dots (QD) are sometimes excited through ultraviolet (UV) irradiation that can lead to autofluorescence (excitation of natural targets, such as elastin, collagen…), photobleaching (destruction of the dye), and luminescence blinking.

Another indirect but efficient way to excite phosphors at low energy for bioimaging is infrared (IR) irradiation, taking advantage of the biological transparency windows: 750–950 nm (BW−1), 1,000–1,450 nm (BW–II), and 1,500–1,700 nm (BW–III). The main advantage is the high signal-to-noise ratio, because biological tissues (containing melanin, hemoglobin and water) absorb less light in these spectral ranges (Shi et al., 2016). Consequently, IR bioimaging does not result in parasitic fluorescence. Moreover, it causes low tissue damage and enables local irradiation along with high penetration depth.

Lanthanide-based upconverting phosphors are based (in the simplest case) on the combination of two absorbed low-energy photons in one of a higher energy, resulting for instance in the absorption of IR wavelengths and emission of visible light (Auzel, 2004). This way, many advantages are conferred to the imaging system (Ajithkumar et al., 2013): the chemical stability and low toxicity of rare-earth compounds, the absence of photobleaching, the low and easy available required energy.

Oxysulfide nanomaterials based on the upconverting properties of lanthanide dopants have been studied as potential upconverting phosphors for biomedical imaging. Ytterbium and erbium co-doped materials are being investigated in detail, but other dopants, such as holmium and thulium have also been reported for upconverting materials.

##### Persistent luminescence

The phenomenon of persistent luminescence is the emission of light by a material after excitation has stopped. It must be distinguished from fluorescence and phosphorescence. Its mechanism is complex and still debated (Jain et al., 2016). In persistent luminescence, the origin of the extended emission in an insulator or semi-conductor is the entrapment of electrons or holes that are progressively released (Leverenz, 1949). Either an electron is trapped in an energy level near the conduction band or a hole is trapped in an energy level near the valence band.

The traps can be point defects with intrinsic defects of the lattice such as vacancies, interstitial defects, antisite defects, or extrinsic defects when doping ions substitute lattice atoms or occupy interstitial sites. Extended defects (dislocations, surface, or grain boundaries) of the lattice can also play the role of traps.

Oxysulfide materials containing titanium and europium have been developed for persistent luminescence. Here, the doping ions substitute the rare-earth of the matrix and correspond to extrinsic defects. Y_2_O_2_S:Ti in 2005 was the first example (Zhang et al., 2005b), but numerous articles focused on the promising properties of Ln_2_O_2_S:Eu^3+^, Mg^2+^, Ti^4+^ (Ln = Gd, Y) which will be named Ln_2_O_2_S:Eu, Mg, Ti for simplification (Mao et al., 2008; Li et al., 2010; Cui et al., 2013a, 2014a; Liu et al., 2014a).

##### Magnetic probes

Because of their remaining *4f* electrons, most of the lanthanide ions present magnetic properties. Lanthanide oxysulfides were found to be paramagnetic in a large range of temperatures, and their magnetic properties at low temperatures were extensively studied (Ballestracci et al., 1968; Quezel et al., 1970; Biondo et al., 2014).

Lanthanides can exhibit high magnetic susceptibility, which is major interest for chemicals that can be injected in a living organism. For instance, Gd^III^ complexes are used as positive contrast agents in magnetic resonance imaging (MRI) due to the *4f*^7^ electronic configuration of the ion (μ = 7.94 μ_B_). The role of a contrast agent is to enhance the MRI signal by locally perturbing the magnetic field. The spin relaxation time of Gd^III^ is long enough to optimize the dipole-dipole interactions of electron and protons (biological tissues, water) in the neighborhood of the contrast agent. The MRI signal is then enhanced by the acceleration of the spin relaxation of the protons caused by these interactions. Gadolinium ions in molecular complexes are toxic because of polarizing effects and competition with calcium. Special hydrosoluble complexes were then developed to prevent the toxicity of Gd^III^ (Tóth et al., 2002).

An alternative to lanthanide complexes is lanthanide nanoparticles. A better detection occurs as the consequence of the concentration of several thousand atoms in a little volume. Iron oxide nanoparticles have been widely studied and used as negative contrast agents, but many artifacts were observed on the resulting images (Bulte and Kraitchman, 2004). Gd_2_O_3_ nanoparticles were found to have a similar or better relaxivity than gadolinium complexes, without the drawbacks of iron oxides. They were then chosen for the precise visualization of locally injected cells (Engström et al., 2006; Petoral et al., 2009).

With doping ions, gadolinium oxide nanoparticles were then applied for bimodal imaging (MRI and luminescence) (Kryza et al., 2011). Because of their very good luminescence properties, similar results are expected for oxysulfide Gd_2_O_2_S nanoparticles. Bimodal agents are especially useful to get various information of the environment of the nanoparticles from the luminescence properties (wavelength, lifetime, and so on) in short times coupled with long term data and precise localization with magnetic resonance imaging (Cherry, 2006). Ajithkumar et al. (2013) demonstrated the possibility of performing multimodal bioimaging using oxysulfide material choosing the Gd_2_O_2_S:Yb, Er phosphor. Besides, Gd_2_O_2_S:Eu micronic particles were used as a colloidal solution for X-ray Luminescence Computed Tomograghy (XLCT), a technique that could be applied *in vivo* (Pratx et al., 2010a,b). Drug delivery can also be tracked *in vivo*. Gd_2_O_2_S:Tb nanoparticles coated with SiO_2_ were employed as radioluminescent markers to evaluate the release of doxorubicin as a function of pH, using X-ray Excited Optical Luminescence (XEOL) (Chen et al., 2013).

##### Catalysis

Recently, sub-micronic powder of Sm_2_Ti_2_O_5_S_2_ was used as a stable photocatalyst for water oxidation and reduction under visible-light irradiation, and this was later further extended to other Ln_2_Ti_2_O_5_S_2_ (Ln = Pr, Nd, Gd, Tb, Dy, Ho, and Er) phases (Ishikawa et al., 2002, 2004). Moreover, because the majority of lanthanides are often restricted to the +III oxidation state, catalysis based on oxido-reduction reactions is not the preferential application of oxysulfide materials. Nevertheless, cerium (Ce^III^ and Ce^IV^) and europium (Eu^II^ and Eu^III^) are notable exceptions. In particular, Ce_2_O_2_S nanoparticles on carbon was tested for oxygen reduction reaction (ORR) (Yang et al., 2017). Also, Eu_2_O_2_S nanoparticles showed catalytic activity for the water-gas shift reaction (reaction of CO with water that yields CO_2_ and H_2_) (Tan et al., 2016). They can also act as a peroxidase mimic for the catalytic oxidation of 3,3′,5,5′-tetramethylbenzidine (TMB) (Ghosh et al., 2016).

#### Synthetic Strategies for Lanthanide Oxysulfide Nanoparticles

##### General pathways toward Ln_2_O_2_S nanoparticles

**Figure 7:**
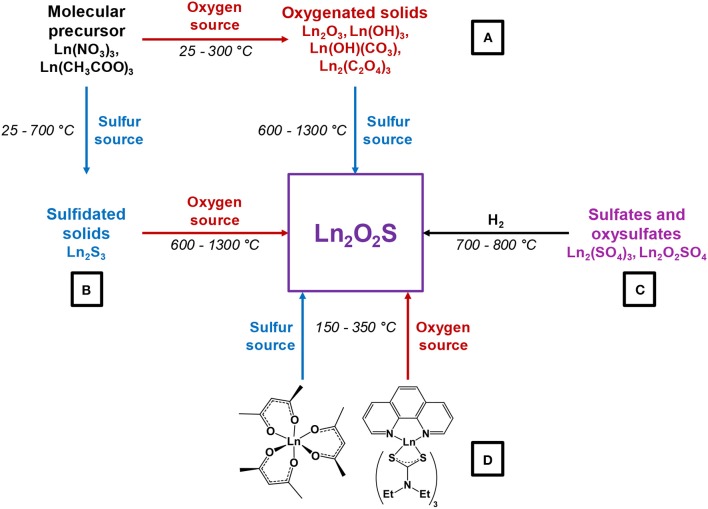
Synthetic strategies toward Ln_2_O_2_S.

Several strategies can be employed to yield oxysulfides nanoparticles. Historically, bulk oxysulfides were formed by partial sulfidation of oxides, oxidation of sulfides or reduction of sulfates (Figure 7). However, solid-gas or solid-solid reactions at high temperatures inevitably lead to sintering and large particles. This should be avoided to control the growth of nanoparticles. Moreover, avoiding sulfates is challenging: their formation is thermodynamically favored.

Four major strategies are employed to yield Ln_2_O_2_S (bulk and nanoparticles). The two first methods are the sulfidation of an oxygenated phase such as an oxide or a hydroxide (Figure 7, pathway A) and the oxidation of sulfides (Figure 7, pathway B). In the latter case, the term “oxidation” names a substitution between sulfur and oxygen and does not imply oxido-reduction processes. This process is challenging: the partial oxygenation of sulfides is hard to control because sulfates are easily formed. To the best of our knowledge, only bulk materials were synthesized this way.

The reduction of sulfates and oxysulfates is also possible (Figure 7, pathway C). It is generally excluded for the formation of nanoparticles as it demands high temperatures (≥800°C). Finally, another way to achieve the synthesis of metal oxysulfides is the co-insertion of oxygen and sulfur. Decompositions of organic precursors containing oxygen or sulfur are especially helpful for this method (Figure 7, pathway D). For syntheses in which oxygen rate has to be finely controlled, inert atmosphere assured by N_2_ or argon is mandatory.

Since 20 years, a broad spectrum of techniques has been developed to yield Ln_2_O_2_S nanoparticles, which remains by far the center of the oxysulfide research. Here, we chose to classify them in three groups mainly depending on the reaction medium: water, organic solvent, and others. As we focused our study on the synthesis of nanomaterials, we excluded the works dealing with particles which were systematically sub-micronic or micronic (>700-800 nm).

##### Typical oxygen and sulfur sources in Ln_2_O_2_S nanoparticles syntheses

*Oxygen source* The oxygen source for the formation of Ln_2_O_2_S nanoparticles highly depends on the synthetic route (Figure 7).

Commonly, in the water-based syntheses, oxygen is brought by hydroxide ions with the precipitation of an intermediate oxygenated phase in basic medium. Oxygen insertion in sulfides Ln_2_S_3_ has never been performed for nanoparticles, to the best of our knowledge. Molecular precursors such as lanthanide formate or lanthanide acetylacetonate contain enough oxygen for the targeted composition. In organic medium, the use of ketones as ligands enables the formation of *in situ* water when an amine is present. The thermal decomposition of single-source precursors with sulfide ligands can be performed in air or pure dioxygen to give Ln_2_O_2_S nanoparticles. In the case of reduction of sulfates and oxysulfates, no additional source of oxygen is required.

**Scheme 1:**
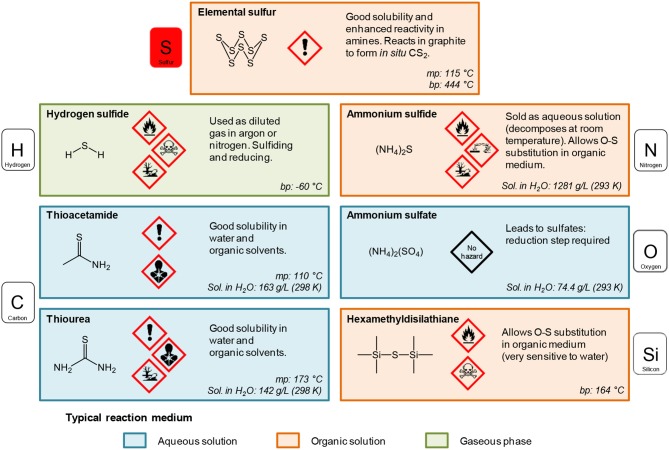
Sulfur sources typically used for the sulfidation processes leading to oxysulfides (mp, melting point; bp, boiling point; Sol., solubility). The first neighbor of sulfur in the molecule is indicated.

*Sulfur sources* (Scheme 1). In water, sulfidation is mainly carried out by solid-gas reaction with H_2_S or *in situ* formed CS_2_ using elemental sulfur heated in graphite or in presence of carbon. Nevertheless, a significant amount of syntheses also use sulfur sources soluble in water, such as thiourea or thioacetamide that initiate the sulfidation process. Elemental sulfur can also be used in organic medium especially dissolved in amines. Recently, substitution of oxygen by sulfur was carried out by ammonium sulfide and hexamethyldisilathiane (HMDTS).

### Exotic Syntheses

Classical nanoparticles syntheses consist in heating hydrophobic or water-soluble inorganic precursors in aqueous or organic media, possibly sealed and/or pressurized and often followed by a thermal treatment which helps sulfidation and/or crystallization. In marge of these techniques, unconventional synthetic methods can be found. They involve unusual solvents, like molten salts, or are performed in uncommon conditions (electrospinning, combustion, and so on). This section describes such syntheses.

#### Boron-Sulfur Method

In 2008, Huang et al. adapted the boron-sulfur method, originally destined to the synthesis of sulfides, to the synthesis of La_2_O_2_S and Nd_2_O_2_S (Huang et al., 2008). In this synthesis, nanowires of the lanthanide hydroxide Ln(OH)_3_ (formed by reaction between Ln(NO_3_)_3_ and KOH) are directly heated in presence of boron and elemental sulfur S_8_ placed in a neighboring crucible. The driving force of the reaction is the strong affinity of boron with oxygen, which leads to the formation of B_2_O_3_ as a by-product.

**Figure 8:**
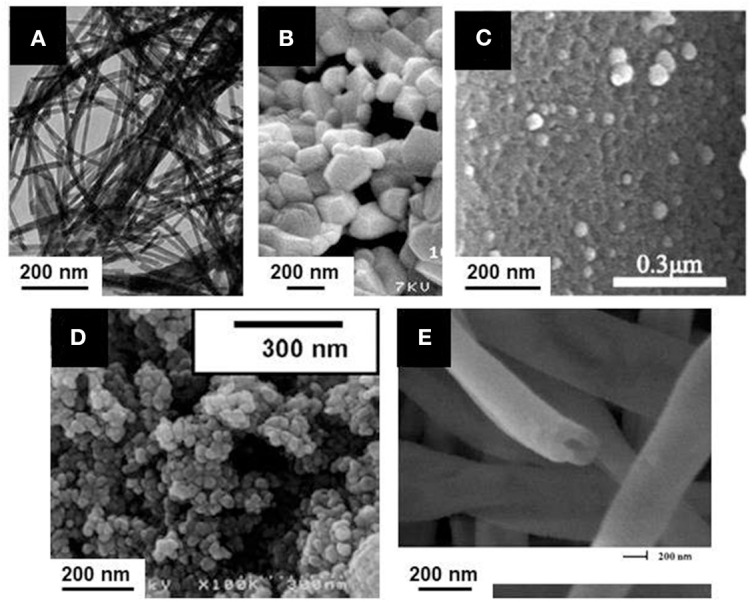
Ln_2_O_2_S nanoparticles obtained from unconventional synthetic methods: **(A)** Boron-sulfur method (La_2_O_2_S:Eu nanowires); Adapted with permission from (Huang et al., 2008), copyright (2008) American Chemical Society. **(B)** Combustion (La_2_O_2_S:Yb, Er nanoparticles); Adapted from Hakmeh et al. (2015) with permission of Elsevier. **(C)** Thermal decomposition of a gel of Pomelo skins (Ce_2_O_2_S nanoparticles supported on carbon); Adapted with permission from Yang et al. (2017), copyright (2017) American Chemical Society. **(D)** Emulsion liquid membrane system (Y_2_O_2_S:Yb, Er nanoparticles); Adapted with permission from Hirai et al. (2002), copyright (2002) American Chemical Society. **(E)** Electrospinning (Y_2_O_2_S: Yb, Er hollow nanofiber). Adapted from Han et al. (2015a) with permission of The Royal Society of Chemistry.

When the reaction is maintained for 24 h at 400°C, LnS_2_ nanowires are obtained. Using shorter reactions times (500°C, 10 min), sulfidation of the wire is partial and Ln_2_O_2_S can be obtained (Figure 8A).

This solid-state reaction preserves the shape of the precursor. Also, it is one of the rare techniques that enable the formation of Ln_2_O_2_S_2_ nanomaterials using in some conditions an excess amount of sulfur compared with the targeted stoichiometry to ensure complete reactions. Nevertheless, only a small quantity of reactants were loaded in the crucible, leading to <15 mg of product per reaction. Also, the remaining species (B_2_O_3_, sulfur in excess) were washed with toxic CS_2_.

#### Combustion Synthesis

In order to get a swift synthesis, Hakmeh et al. (2015) developed a combustion synthesis by mixing lanthanide nitrates [La(NO_3_)_3_, Er(NO_3_)_3_ and Yb(NO_3_)_3_] with thioacetamide in ethanol. The precursors were rapidly inserted in a furnace at 500°C. Two successive flames evidenced first the ignition of ethanol, then the exothermic decomposition of the organic compounds, leading to an increase of the temperature and eventually to the formation of particles. A post-treatment at high temperature was also necessary (H_2_S in N_2_, 2 h, 1,000°C) and resulted in large particles with a typical size around 300-500 nm (Figure 8B).

#### N,S Dual Doped Carbon Supported Ce_2_O_2_S

Recently, an original catalyst for oxygen reduction reaction (ORR) was obtained by using the thermal decomposition of a vegetal, which provides the carbon support for the inorganic catalyst (Yang et al., 2017). Cerium nitrate [Ce(NO_3_)_3_] was dissolved in water along with thiourea and then pomelo skins were added to the solution in order to form a gel. After drying, the gel was annealed at 900-950°C for 2 h to get Ce_2_O_2_S supported on carbon doped by nitrogen and sulfur. When the reaction temperature was set to 850 or 1,000°C, the reaction led to the formation of CeO_2_. The TEM observation of the catalyst shows 50-100 nm crystals of Ce_2_O_2_S disseminated on the surface of the samples (Figure 8C). The porous structure, inherited from the pomelo precursor and the oxygen vacancies evidenced by the authors make this material suitable for the ORR.

#### Emulsion Liquid Membrane System (ELM)

Emulsion Liquid Membrane System (ELM) employs a water-in-oil-in-water (W/O/W) double emulsion. Originally, ELM was applied to separate metals. Here, the double emulsion is used for the formation of doped yttrium and gadolinium oxalates. These intermediates are converted to oxysulfides, Y_2_O_2_S:Yb, Er, and Gd_2_O_2_S:Eu, by a solid-state reaction with sulfur vapor (Hirai et al., 2002; Hirai and Orikoshi, 2004). Typically, a first emulsion is obtained by mechanical agitation of an organic phase containing kerosene with bis(1,1,3,3-tetramethylbutyl)phosphinic acid (DTMBPA) (or 2-methyl-2-ethylheptanoïc acid, VA-10) as extractant and sorbitan sesquioleate as surfactant and an aqueous phase containing oxalic acid. This emulsion is then added to the external water phase which contains the metal ions (chloride or nitrates) and the double emulsion is produced by mechanical stirring. The oxalate compounds are thus produced at ambient temperature, and the system is demulsified using ethylene glycol. Oxysulfides nanoparticles of 50-100 nm are then obtained by annealing the powders at 600-1,000°C in sulfur vapor generated at 200°C by elemental sulfur and carried by a N_2_ flow (Figure 8D).

#### Synthesis in Molten Sodium Chloride

The synthesis in molten salts is an emerging technique which consists in the use of one or several salts as solvents for an inorganic reaction. An eutectic mixture can even be used to benefit from a lower melting point. Molten salts are typically suitable for reaction temperatures between 300 and 1,000°C, which enable the formation of nanoparticles while avoiding their sintering (Portehault et al., 2011; Gouget et al., 2017). After cooling, the particles are obtained in a matrix composed by the salts that are washed with water or alcohols.

Molten sodium chloride (melting point: 801°C) was chosen for the one-pot synthesis of Y_2_O_2_S:Eu. Y(NO_3_)_3_, Eu(NO_3_)_3_ and NaOH were mixed and stirred before the addition of NaCl, S_8_ and a surfactant (Wang et al., 2014). After grinding, the mixture was heated to 850°C in a CO atmosphere for 4 h, and then cooled and washed.

**Scheme 2:**
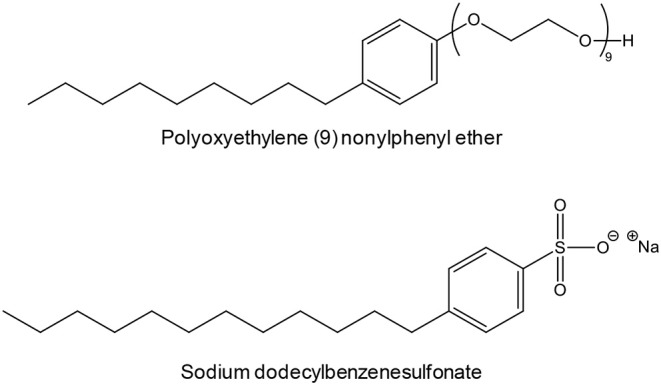
Examples of surfactants employed by Wang et al. to synthesize Y_2_O_2_S:Eu in molten NaCl (Wang et al., 2014).

Depending on the surfactant, the particles were either sub-micrometric or nanoscaled, but the morphology was quite irregular and the size polydisperse in all cases. For instance, polyoxyethylene (9) nonylphenyl ether (Scheme 2) gave 150-250 nm particles while sodium dodecylbenzenesulfonate (Scheme 2) gave 0.5-1.5 μm particles.

#### Composite-Hydroxide-Mediated Method

**Figure 9:**
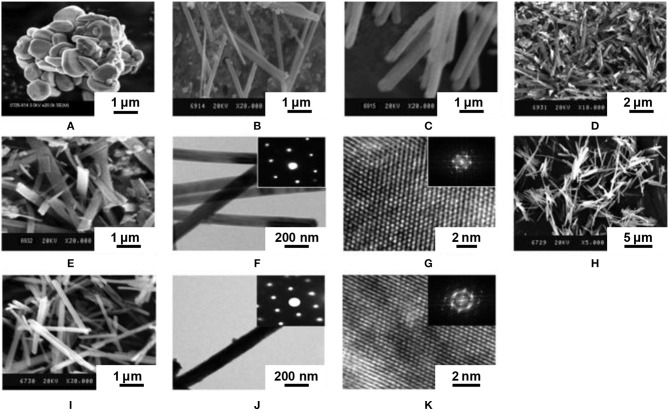
Europium-doped yttrium oxysulfide nanoparticles with different morphologies obtained via the composite-hydroxide-mediated method and their precursors. **(A)** SEM image of bulk Y_2_O_2_S:Eu. **(B)** SEM image of Y(OH)_3_ nanobelts. **(C)** SEM image of Y(OH)_3_ nanorods. **(D)** SEM image of Y_2_O_2_S:Eu nanobelts. **(E)** SEM image of Y_2_O_2_S:Eu nanorods. **(F)** TEM and **(G)** HRTEM images of Y_2_O_2_S:Eu nanobelts. **(H)** High-magnification SEM image of Y_2_O_2_S:Eu nanobelts. **(I)** High-magnification SEM image of Y_2_O_2_S:Eu nanorods. **(J)** TEM and **(K)** HRTEM images of Y_2_O_2_S:Eu nanorods. Insets are corresponding SAED patterns. Adapted from Thirumalai et al. (2011a), copyright (2011) The Institution of Engineering and Technology.

The composite-hydroxide-mediated method is also a synthesis in molten salts, but with hydroxides. Thirumalai et al. (2011a) adapted this method to the synthesis of Eu-doped yttrium oxysulfide by heating concentrated yttrium acetate Y(CH_3_COO)_3_ in an eutectic mixture of NaOH and KOH in an autoclave. As the eutectic of the mixture is 165°C, the autoclave was heated at 200°C to yield Y(OH)_3_ nanobelts (48 h) and nanorods (24 h) (Figure 9).

**Figure:**
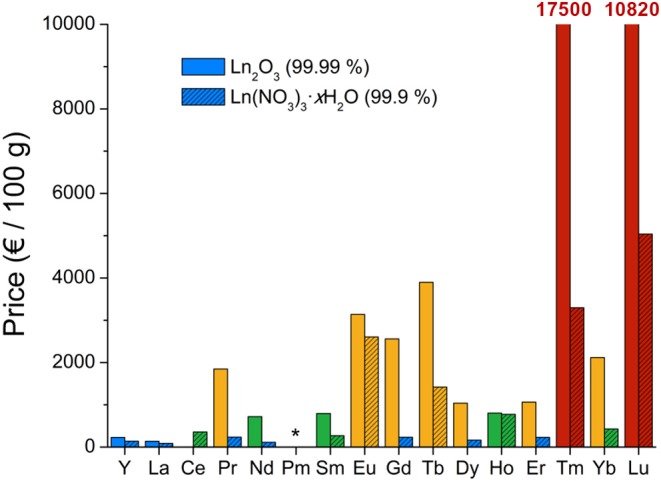


Europium and S_8_ were then mixed with the Y(OH)_3_ nanomaterial at 70-80°C and underwent an undescribed sulfidation process. In any case, the product was then annealed at 600°C for 2 h in an (Ar or N_2_)/sulfur atmosphere to form Y_2_O_2_S:Eu. Interestingly, the final product retained the morphology of the Y(OH)_3_ precursor. On the other hand, the step where the product was sulfidated was particularly unclear here, as three sulfidation processes are mentioned.

#### Electrospinning

Electrospinning is based on the application of a high potential difference between a polymer solution or a polymer melt and a collector. The electrical field creates charged threads that can be assembled depending on the experimental parameters such as tension, temperature, relative humidity (RH), concentration of the precursors, viscosity, distance between capillary screen and collection screen, etc.

Lanthanide nitrates Y(NO_3_)_3_, Yb(NO_3_)_3_, and Er(NO_3_)_3_ and polyvinyl pyrrolidone (PVP) were dissolved in DMF and stirred 8 h (Han et al., 2015a). Fibers were produced by electrospinning. They were annealed twice: (i) at 700°C for 8 h under air to get Y_2_O_3_:Tb, Er fibers and (ii) at 800°C for 4 h in a CS_2_ atmosphere (obtained by heating S_8_ in presence of carbon) to yield Y_2_O_2_S:Yb, Er hollow nanofibers (Figure 8E). The same strategy was used to yield Y_2_O_2_S:Er hollow nanofibers (Han et al., 2015b). With slightly different electrospinning parameters, full nanofibers of Y_2_O_2_S:Yb, Er with a diameter comprised between 80 and 140 nm were obtained and studied by Lu et al. (2015).

#### Anodic Aluminum Oxide Template

In 2013, Cui et al. (2013a,b) elaborated a synthesis for doped oxysulfide nanoarrays using an anodic aluminum oxide template (AAO). A nitrate solution obtained by dissolution of Y_2_O_3_, Eu_2_O_3_, and Mg(OH)_2_·4MgCO_3_·2H_2_O in hot HNO_3_ (65%) was diluted by ethanol. Titanium doping was then obtained by adding the reaction product of Ti(OBu)_4_ with acetylacetone. The pH was adjusted to 1 with HNO_3_. The sol was eventually obtained by evaporation at 80-90°C. The AAO template was dipped in the sol, dried, calcined at 600°C for 2 h and etched by NaOH (2.0 M) to give Y_2_O_3_:Eu, Mg, Ti nanoarrays. The whole process involved numerous steps and the resulting nanoarrays had to be sulfurated to Y_2_O_2_S:Eu, Mg, Ti using S_8_ in graphite at 850°C sharp (Cui et al., 2013a). Lower and higher temperatures were indeed not adequate: they resulted, respectively, in uncomplete sulfidation or oxide formation. Besides, an optimal concentration of europium dopant for the luminescence properties was determined (6.5 mol% Eu vs. Y) (Cui et al., 2013b).

### Water-Based Syntheses

In the following syntheses, the reaction medium is water. It is an available, green, and ideal solvent for the dissolution of numerous metallic precursors, especially nitrates and chlorides.

Water also brings two main advantages: first, the availability of lanthanide precursors, and especially nitrates (that can be prepared from oxides in HNO_3_) and water-soluble sulfur sources (thioacetamide, thiourea, ammonium sulfide, sodium sulfide, and so on; see Scheme 1); second, the substantial knowledge on inorganic polymerization in water. So far, in more than 90% of the articles dealing with Ln_2_O_2_S nanoparticles, the desired feature of the material was luminescence. Luminescence is due to a controlled doping of the oxysulfide phase (Ln^1^_2_O_2_S:Ln^2^, M^3^, M^4^) that is achieved by co-precipitation of the main cation (Ln^1^) with the cations that trigger the luminescence and influence its properties (Ln^2^, and possibly M^3^, M^4^,…).

Water is however limiting metal oxysulfide synthesis by its relatively low boiling point. Even hydrothermal syntheses with autoclaves do not provide enough energy to obtain crystalline oxysulfide nanoparticles. In general, syntheses lead to an intermediate nanoscaled phase (which sometimes already contains sulfur) that is subsequently fully converted in oxysulfide nanoparticles with a solid-gas sulfidation (Figure 7). This last step remains an important drawback. It requires relatively high temperatures for nanoparticles synthesis (typically between 600 and 1,100°C) and a large excess of inert gas and sulfur which is often present under the active but toxic gaseous forms of H_2_S or CS_2_. Also, it can affect the morphology of the solid by sintering or degradation of the desired phase.

The high-temperature sulfidation step remains the most challenging process here, but can be useful for other features. For luminescence purposes, the energy provided during the thermal treatment gives better-crystallized nanoparticles that present better photoluminescence properties. Moreover, doping ions can be inserted during this step.

#### Gelatin-Templated Synthesis

Reported in 2008 by Liu et al., this synthesis stands out through the original use of gelatin and the way the oxysulfide phase is obtained (Liu et al., 2008).

First, the appropriate amounts of lanthanum, terbium, and europium nitrates obtained from dissolution of La_2_O_3_, Tb_4_O_7_, and Eu_2_O_3_ in nitric acid are mixed and heated with gelatin at 80°C in H_2_O. The obtained translucent gelatin sol turns into a gel at 0°C. Small pieces of the gel are soaked into NH_3_·H_2_O and La(OH)_3_:Eu, Tb precipitates inside the gel. Violent stirring can then turn the gel into sol again, and (NH_4_)_2_(SO_4_) is added in stoichiometric amount. After drying and annealing at 500°C for 2 h in air, a powder of oxysulfate La_2_O_2_SO_4_:Eu, Tb nanoparticles is formed. The oxysulfate nanoparticles are then converted to oxysulfide nanoparticles by solid-gas reaction using H_2_ as reducing gas (700-800°C, 2 h).

The pathway of oxysulfate reduction is quite rare in the oxysulfide nanoparticles literature, as it often requires high temperatures and long reaction times. Here, the nanoparticles however keep a reasonable 50 nm diameter. On the other hand, this synthesis comprises a myriad of steps, generates two intermediary phases and requires two heat treatments above 500°C.

#### Sol-gel Polymer Thermolysis

This strategy is based on the elaboration of an organic network in which the inorganic nanoparticles nucleate and grow in a controlled way. The network is then burnt to free the nanoparticles. It is analogous to the Pechini method used for oxide synthesis for which a tridimensional polyester network is elaborated by reaction of trisodium citrate and ethylene glycol for instance (Pechini, 1967).

Dhanaraj et al. published in 2003 a first version of a sol-gel polymer thermolysis strategy to yield Y_2_O_2_S:Eu nanoparticles (Dhanaraj et al., 2003). Y(NO_3_)_3_ and Eu(NO_3_)_3_ were obtained from the corresponding oxides. Urea, formaldehyde and elemental sulfur were then added and the network was formed at 60°C. By condensation of urea and formaldehyde along with water evaporation, a gel was obtained. After thermolysis at 500°C in sulfidating atmosphere, Y_2_O_2_S:Eu nanoparticles were formed. Based on the XRD pattern, the product was not pure (small peaks of impurities). Despite the treatment at 500°C, the nanoparticles were quite small (around 30–50 nm) but presented an unclear morphology and aggregation. The work of Dai et al. in 2008 on La_2_O_2_S:Eu which deals with the effects of Eu^3+^ concentration on the photoluminescence is based on the same synthetic route (Dai et al., 2008).

One year later, Dhanaraj et al. published a second version of the protocol that led to hexagonal nanoplates with a size between 7 and 15 nm, tunable via the reactants concentrations (Dhanaraj et al., 2004). The thermolysis process was divided in two steps: first, the sol/network solid was heated at 500°C for 2 h to get Y_2_O_3_:Eu nanoparticles, and was subsequently digested by a thiosulfate solution. After water evaporation, a second thermal treatment at 500°C (1 h) burnt the mixture to yield Y_2_O_2_S:Eu nanoparticles. The authors did not obtain a pure product yet, based on XRD analysis, but this time they identified sodium polysulfides as side-products. Later, Thirumalai and Nakkiran reused this strategy, succeeded in washing the by-products (Thirumalai et al., 2007) and deeply investigated the nanoparticles: optical (Thirumalai et al., 2007, 2008a) and electronic properties (Thirumalai et al., 2008a) were discussed as well as the photo-assisted relaxation of surface states (Nakkiran et al., 2007).

#### Syntheses in Water at Atmospheric Pressure

Because of the attractiveness of luminescent water-dispersible nanoparticles, the pursuit of doped oxysulfide nanoparticles led to the publication and the refinement of synthetic strategies in water. However, the reported syntheses illustrate the complexity of obtaining oxysulfides at low temperatures in water: most often, the authors choose to precipitate an unsulfurated intermediary doped phase [Ln(OH)_3_, Ln(OH)(CO_3_) for instance] that can be amorphous or not. Thus, the syntheses presented in this section are worthwhile for oxide-, hydroxide-, or hydroxycarbonate-based nanomaterials. The intermediate nanoparticles are then sulfidated, most often with a solid-gas or alternatively with a solid-solid reaction.

Interestingly, the conditions for lanthanide oxysulfide nanoparticles syntheses in water are majorly optimized on Gd_2_O_2_S and Y_2_O_2_S because of their well-known luminescent properties and also maybe for the relatively low price of the related precursors (Table 2).

**Table 2:** Lanthanide oxide and nitrate prices (October 2018).

**Element**	**Ln_**2**_O_**3**_** **€/100 g^**a**^**	**Ln_**2**_O_**3**_** **99.99%, €/100 g^**b**^**	**Ln(NO_**3**_)_**3**_**·***x*H_**2**_O** **99.9%, €/100 g^**b**^**	**Cost^**c**^**
Y	0.26	230	140 (99.8%)	•
La	0.16	139	89	•
Ce	/	/	359	•
Pr	5.08	1,850 (99.9%)	236 (99.99%)	•
Nd	4.04	722	113	•
Sm	0.17	796	271	•
Eu	3.71	3,140	2,608	•
Gd	1.72	2,560	235	•
Tb	38.0	3,900	1,420	•
Dy	15.9	1,040	166	•
Ho	/	806 (99.9%)	776	•
Er	2.01	1,064	233	•
Tm	/	17,500	3,300	•
Yb	/	2,120	430	•
Lu	/	10,820	5,040	•

a Prices on Shanghai Metal Market. Original prices units are RMB/mt or RMB/kg and were converted.

b Prices on Merck on October 29th 2018 for France.

c From more affordable to more expensive: blue-green-yellow-red.

^*^no stable isotope.

##### Urea-based syntheses

**Scheme 3:**
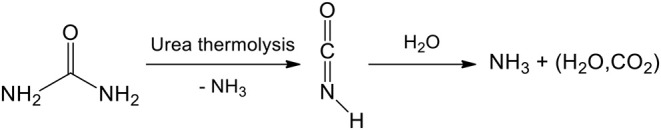
Urea decomposition in water. Isocyanic acid is slowly obtained by urea thermolysis and ammoniac release. A second ammoniac molecule is released by hydrolysis which gives aqueous carbon dioxide “H_2_CO_3_.”

*Decomposition of urea in water.* Generally, the precipitation of the lanthanide salts is performed via the basification of the reaction medium. Thus, a significant amount of research has focused on the cheap, safe, highly available, and water-soluble urea. Urea is indeed known to decompose in ammonia [pKa(${\text{NH}}_{4^{+}}$,NH_3_) = 9.25] and aqueous carbon dioxide which can carbonate aqueous lanthanide species (Scheme 3).

The concomitant release of ammonia and aqueous carbon dioxide is used in particular for the precipitation of lanthanide hydroxycarbonates Ln(OH)CO_3_ that turned out to be a suitable precursor of lanthanide oxysulfide nanoparticles. In the absence of sulfur source, further decomposition of this intermediate leads instead to lanthanide oxide. This was demonstrated in the pioneering work of Matijević and Hsu (1987) in the context of the fabrication of well-calibrated lanthanide colloids.

*Syntheses with urea in water.* The first aqueous synthesis of oxysulfide nanoparticles was reported by Kawahara et al. (2006; Table 3). Using yttrium and europium nitrates Y(NO_3_)_3_ and Eu(NO_3_)_3_ along with urea, an europium-doped hydroxide precursor Y(OH)_3_:Eu was obtained by heating the mixture possibly in the presence of a glycol (ethylene glycol, propylene glycol, or hexamethylene glycol). The isolated powder of Y(OH)_3_:Eu was then heated between 800 and 1,200°C with Na_2_CO_3_ and sulfur to create a sulfidating vapor and yield Y_2_O_2_S:Eu nanoparticles. XRD showed that the crystalline phase was pure Y_2_O_2_S. The obtained nanoparticles were facetted crystals of 100-300 nm length. Above 1,100°C, sintering made the particles sub-micrometric (≥600 nm).

**Table 3:** Precipitation from aqueous solutions at atmospheric pressure.

**References** **Phase**	**Metal sources**	**Precipitation step(s)**	**Annealing step(s)**	**Intermediary phase(s)**	**Final Morphology (Final size)**
Kawahara et al. (2006) Y_2_O_2_S:Eu	Y(NO_3_)_3_ Eu(NO_3_)_3_	1/Urea, glycol 100°C, 5 h	2/S_8_, Na_2_CO_3_ 800–1,200°C, 2 h	1/Y(OH)_3_:Eu	Nanocrystals (100–300 nm for T ≤ 1,100°C)
Xing et al. (2009) Y_2_O_2_S:Yb,Ho Pang et al. (2010) Y_2_O_2_S:Yb,Ho@SiO_2_-APTES Bakhtiari et al. (2015) Y_2_O_2_S:Eu	Y(NO_3_)_3_ Yb(NO_3_)_3_ Ho(NO_3_)_3_	1/Urea 82°C 2/Aging r.t., 48 h	3/Air, 600°C, 1 h 4/S vapor (S_8_ at 400°C) Ar, 800°C, 1 h	2/Y(OH)CO_3_:Yb,Ho^a^ 3/Y_2_O_3_:Yb,Ho (after annealing in air)	Nanospheres (Ø ≈ 70 nm)
Luo et al. (2009) Y_2_O_2_S:Yb,Ho	Y(NO_3_)_3_ Yb(NO_3_)_3_ Ho(NO_3_)_3_	1/Urea (oleic acid) 82°C 2/Aging r.t., 48 h	3/Air, 600°C, 1 h 4/S vapor (S_8_ at 400°C) Ar, 550–600°C, 1 h	2/Y(OH)CO_3_:Yb,Ho^a^ 3/Y_2_O_3_:Yb,Ho	Nanospheres (Ø ≈ 50 nm)
Ai et al. (2010b) Y_2_O_2_S:Eu,Mg,Ti	Y(NO_3_)_3_ Eu(NO_3_)_3_	1/Urea 90°C, 3 h	2/Air, 700°C, 2 h 3/S_8_ in graphite (CS_2_) 800°C, 4 h 4/TiO_2_, Mg(OH)_2_·4Mg(CO_3_)·6H_2_O 1,100°C, 4 h	2/Y_2_O_3_:Eu	Hollow submicrospheres (Ø = 350–400 nm)
Ai et al. (2010a) Y_2_O_2_S:Eu,Mg,Ti	Y(NO_3_)_3_ Eu(NO_3_)_3_	1/Urea 90°C, 2 h	2/S_8_ in graphite (CS_2_) 800°C, 4 h 3/TiO_2_, Mg(OH)_2_·4Mg(CO_3_)·6H_2_O 1,100°C, 4 h	1/Y(OH)CO_3_:Eu	Nanospheres (Ø = 100–150 nm)
Fu et al. (2010) Y_2_O_2_S:Yb,Ho	Y(NO_3_)_3_ Yb(NO_3_)_3_ Ho(NO_3_)_3_	1/Na_2_CO_3_ PEG 4000	2/Air, 600°C, 1 h 3/S vapor (S_8_ at 400°C) Ar, 800°C, 1 h	1/Y(OH)CO_3_:Yb,Ho^a^ 2/Y_2_O_3_:Yb,Ho	Nanocrystals (30–100 nm)
Osseni (2012) Gd_2_O_2_S:Eu Dy_2_O_2_S:Eu Ho_2_O_2_S:Eu	Gd(NO_3_)_3_ Dy(NO_3_)_3_ Ho(NO_3_)_3_ Eu(NO_3_)_3_	1/Urea EtOH 85°C	2/Ar/H_2_S (83/17 v/v) 750°C, 90 min 3/Ar, 850°C, 4 h	1/Gd(OH)CO_3_·H_2_O:Eu Dy(OH)CO_3_·H_2_O:Eu Ho(OH)CO_3_·H_2_O:Eu	Nanospheres (Ø = 60 nm for Gd, 170 nm for Dy, 53 nm for Ho)
Osseni et al. (2011) Gd_2_O_2_S:Eu@mSiO_2_ Gd_2_O_2_S:Eu@SiO_2_-APTMS	Gd(NO_3_)_3_ Eu(NO_3_)_3_	1/Urea EtOH 85°C	2/Ar/H_2_S (83/17 v/v) 750°C, 90 min 3/Ar, 850°C, 4 h	1/Gd(OH)CO_3_·H_2_O:Eu	Nanospheres, tunable diameter with EtOH volume (Ø = 100–250 nm) Silica coating: 10 nm
Yan et al. (2013a) Ln_2_O_2_S:Tb (Ln = Gd, Y) Yan et al. (2013b) Y_2_O_2_S:Tb,Er	Ln(NO_3_)_3_ Tb(NO_3_)_3_ (Er(NO_3_)_3_)	1/Urea ~100°C, 1 h	2/Na_2_CO_3_, S_8_, Ln_2_O_3_ 900°C, 1 h	1/Ln(OH)CO_3_·H_2_O:Tb or Y(OH)CO_3_·H_2_O:Tb,Er	Nanocrystals (50–200 nm)
Hernández-Adame et al. (2014) Gd_2_O_2_S:Tb	Gd(NO_3_)_3_ Tb(NO_3_)_3_	1/Urea	2/Air, 800°C, 2 h 3/S vapor (S_8_ at 900°C) N_2_, 900°C, 3 h	1/Gd(OH)CO_3_·H_2_O:Tb 2/Gd_2_O_3_:Tb	Good conditions give nanospheres (Ø = 100 nm)
Tian et al. (2015) Y_2_O_2_S:Yb,Er	Y(NO_3_)_3_ Yb(NO_3_)_3_ Er(NO_3_)_3_	1/NH_4_HCO_3_ NH_3_·H_2_O r. t.	2/S vapor (S_8_ at 400°C) N_2_, 900°C, 1 h	1/Y(OH)_x_(CO_3_)_y_:Yb,Er	Aggregated nanocrystals (30 nm)
Cichos et al. (2016) Gd_2_O_2_S:Eu	Gd(NO_3_)_3_ Eu(NO_3_)_3_	1/Urea ~100°C (a) 2 h, (b) 24 h	2/S_8_, Ar 950°C, 1 h	1/(a) amorphous (b) Gd(OH)CO_3_:Eu	(a) Nanospheres (Ø ≈ 130 nm) (b) Microcrystals (≈ 1 μm)
Bagheri et al. (2016) Gd_2_O_2_S:Pr	Gd(NO_3_)_3_ Pr(NO_3_)_3_	1/Urea ~100°C, 1 h	2/Air, 600°C, 1 h 3/S_8_, 900°C, 1 h	1/Gd(OH)CO_3_·H_2_O:Pr^a^	Nanospheres (Ø = 25–80 nm)
Tian et al. (2017) Y_2_O_2_S:Er@Y_2_O_2_S:Yb,Tm	Y(NO_3_)_3_ Er(NO_3_)_3_ Yb(NO_3_)_3_ Tm(NO_3_)_3_	1/Urea 82°C 2/Aging r.t., 48 h	3/S vapor (S_8_ at 800°C) Ar, 800°C, 40 min	2/Y_2_O_3_:Er 2′/Y_2_O_3_Er@Y_2_O_3_:Yb,Tm	Aggregated crystals (50–150 nm)

a *Intermediary phase deducted from later works*.

**Figure 10:**
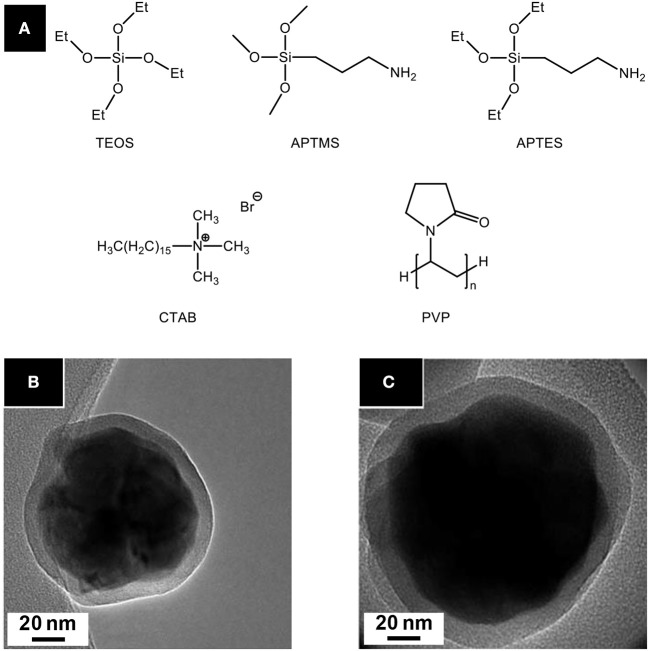
**(A)** Precursors and additives commonly used for nanoparticles silica coating. Tetraethylorthosilicate (TEOS) is used as silica precursor; 3-aminopropyltrimethoxysilane (APTMS), and 3-aminopropyltriethoxysilane (APTES) are rather employed for silica functionalization. TEM micrographs of Gd_2_O_2_S:Eu@SiO_2_-APTMS **(B)** and Gd_2_O_2_S:Eu@mSiO_2_ **(C)** nanoparticles from Osseni et al. (mSiO_2_ stands for mesoporous silica). Adapted from Osseni et al. (2011) with permission of The Royal Society of Chemistry.

Xing et al. (2009) then developed an inspiring but complex protocol to synthesize Y_2_O_2_S:Yb, Ho upconversion nanoparticles (Xing et al., 2009). A solution of lanthanide nitrates Y(NO_3_)_3_, Yb(NO_3_)_3_, and Ho(NO_3_)_3_ and a solution of urea were separately prepared. The latter solution was added to the first that had been pre-heated at 60°C and the mixture was then heated at 82°C. After cooling and aging during 48 h, a white amorphous precipitate [likely Y(OH)CO_3_] (Tian et al., 2017) was dried and converted to Y_2_O_3_:Yb, Ho via calcination (600°C, 1 h, air). Then, the oxide was sulfidated at 800°C for 1 h with a sulfur vapor created by S_8_ at 400°C and conveyed by an argon flow. It enabled the formation of size-monodisperse and non-aggregated nanoparticles with an average diameter of *ca* 80 nm. The diameter could also be tuned by adjusting the reaction time (aging step). Several works are based on Xing's synthesis with slight modifications. Luo et al. added a small amount of oleic acid in the urea mixture and performed the sulfidation at only 600°C to form the same Y_2_O_2_S:Yb, Ho nanoparticles (Luo et al., 2009). In the same group, Pang et al. (2010) reported additional reactions that coated the nanoparticles with functionalized silica using a derived Stöber process with polyvinylpyrrolidone (PVP), aqueous ammonia, teraethylorthosilicate (TEOS), and aminopropyltriethoxysilane (APTES) in a second step (Figure 10). Sulfidation of hydrated Ln(OH)CO_3_:Eu^3+^ nanoparticles (Ln = Gd, Dy, Ho) was alternatively performed under a flow of H_2_S at 750°C for 90 min followed by an annealing under Ar at 850°C for 4 h (Verelst et al., 2010; Osseni, 2012) This constitutes the sole reported route to Dy_2_O_2_S and Ho_2_O_2_S nanoparticles, to the best of our knowledge.

**Figure 11:**
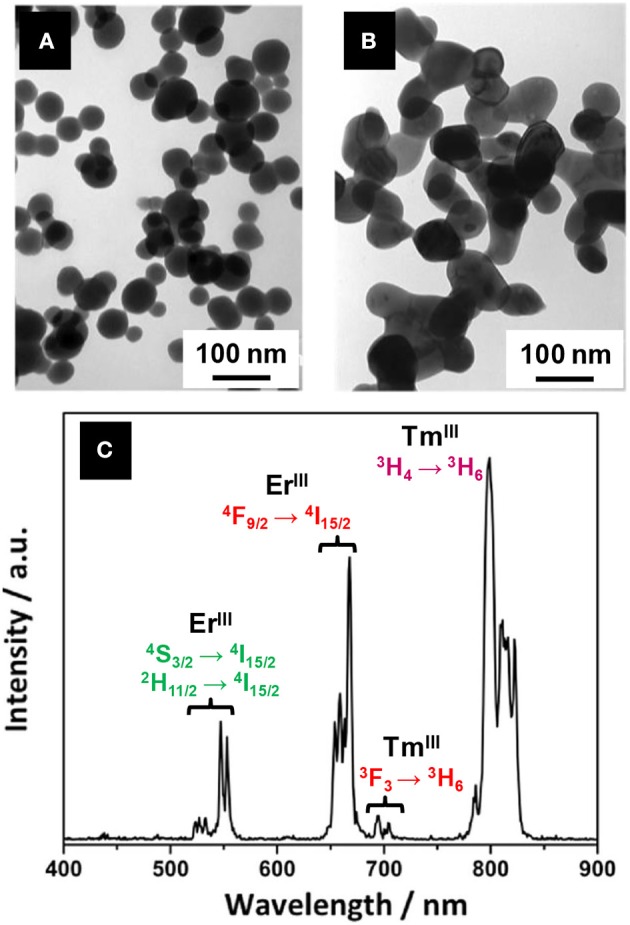
TEM micrographs of Y_2_O_3_:Er **(A)** and Y_2_O_2_S:Er@Y_2_O_2_S:Yb,Tm nanoparticles **(B)** synthesized by Tian et al. **(C)** Luminescence spectrum of Y_2_O_2_S:Er@Y_2_O_2_S:Yb, Tm nanoparticles under 1,550 nm excitation that exhibits the multicolor fluorescence of the core-shell nanoparticles (in the near-infrared region, between 780 and 830 nm, the ^4^I_9/2_→^4^I_15/2_ transition of Er^III^ was proven to play a minor role). Adapted from Tian et al. (2017) with permission of Elsevier.

Also based on Xing's work, Bakhtiari et al. (2015) later studied the effect of europium concentration on Y_2_O_2_S:Eu nanoparticles size and luminescence. Very recently, Tian et al. succeeded in forming upconverting core-shell nanoparticles Y_2_O_2_S:Er@Y_2_O_2_S:Yb,Tm by applying Xing's method twice to form the oxide-oxide compound Y_2_O_3_:Er@Y_2_O_3_:Yb,Tm as an intermediate (Tian et al., 2017). Solid-gas reaction with sulfur vapor at 800°C finally provided the oxysulfide nanoparticles. After the shell formation, Y_2_O_3_:Er@Y_2_O_3_:Yb,Tm nanoparticles were well-separated (Figure 11A). After sulfidation, the nanoparticles were aggregated because of sintering (Figure 11B). Nevertheless, the shell prevented the quenching of the Er^III^ luminescence and multicolor fluorescence was achieved thanks to Er^III^/Tm^III^ co-doping (Figure 11C).

Y_2_O_2_S:Eu, Mg, Ti nanoparticles were also synthesized for persistent luminescence applications by Ai et al. (2010a). Y(OH)CO_3_:Eu was obtained by heating a mixture of Y(NO_3_)_3_, Eu(NO_3_)_3_, and urea at 90°C for 2 h. The final product is obtained by a two-step thermal treatment developed by Li et al. (2009), It involves first S_8_ in graphite at 800°C for 4 h, which creates *in situ* reactive CS_2_, and then solid-solid reaction with doping solids (here Mg(OH)_2_·4MgCO_3_·6H_2_O and TiO_2_). The same year, Ai et al. (2010b) presented an original morphology for the same phase. Hollow submicrospheres were obtained using templating 350-400 nm carbon submicrospheres obtained by hydrothermal glucose decomposition (autoclave, 160°C, 9 h). Before sulfidation and Mg/Ti doping, Y_2_O_3_:Eu was obtained when removing carbon by thermal treatment at 700°C (2 h, air).

In 2011, Osseni et al. reported the first synthesis of Gd_2_O_2_S:Eu nanoparticles starting from nitrates and urea in a water/ethanol mixture (H_2_O/EtOH = 80/20 *v*/*v*) (Osseni et al., 2011). After dissolution, the reactants were heated to 85°C to form a doped hydroxycarbonate precursor Gd(OH)CO_3_·H_2_O:Eu. After isolation and drying, a heat treatment in two steps was performed. First, sulfidation was performed by Ar/H_2_S at 750°C for 90 min and then the nanoparticles were maintained at 850°C for 4 h under argon atmosphere only. The final nanoparticles were crystalline and spherical. Diameter was tunable by varying the H_2_O/EtOH ratio and reaction time. Interestingly, two techniques of deposition of silica on the nanoparticles were presented. The shell was either formed of mesoporous silica using TEOS and cetyltrimethylammonium bromide (CTAB) or functionalized by a silica/APTMS shell using TEOS and 3-aminopropyltrimethoxysilane (APTMS). In particular, mesoporous silica was found to enhance the luminescence properties of the nanoparticles. Multimodal imaging was recently applied using these Gd_2_O_2_S:Eu nanoparticles (Santelli et al., 2018).

A slightly different strategy, close to the work of Xing et al. on yttrium, was adopted in 2013 by Yan et al. (2013a) for the formation of terbium-doped oxysulfide nanoparticles of gadolinium and yttrium. Tb(NO_3_)_3_ and Gd(NO_3_)_3_ were dissolved in water around 100°C, and then urea was added. After filtration and drying, Gd(OH)CO_3_·H_2_O:Tb was obtained. The sulfidation process was quite complex: the precursor is mixed with Na_2_CO_3_ and sulfur but is also covered by a second mixture composed of Gd_2_O_3_, Na_2_CO_3_, and S_8_. The bottom layer was washed in hot water and filtrated after being fired at 900°C for 1 h. The crystalline phases Gd_2_O_2_S or alternatively Y_2_O_2_S were pure (based on XRD) and the polydispersity of the diameter was significant (average diameter around 100–120 nm). Yan et al. also studied the role of the doping ions in the luminescence mechanism of Y_2_O_2_S:Tb, Er nanoparticles (Yan et al., 2013b). In 2016, Bagheri et al. fabricated a scintillator screen composed of Gd_2_O_2_S:Pr nanoparticles synthesized via a similar nitrate/urea reaction (Bagheri et al., 2016). However, the sulfidation process is a solid-solid reaction with S_8_ at 900°C for 1 h.

**Figure 12:**
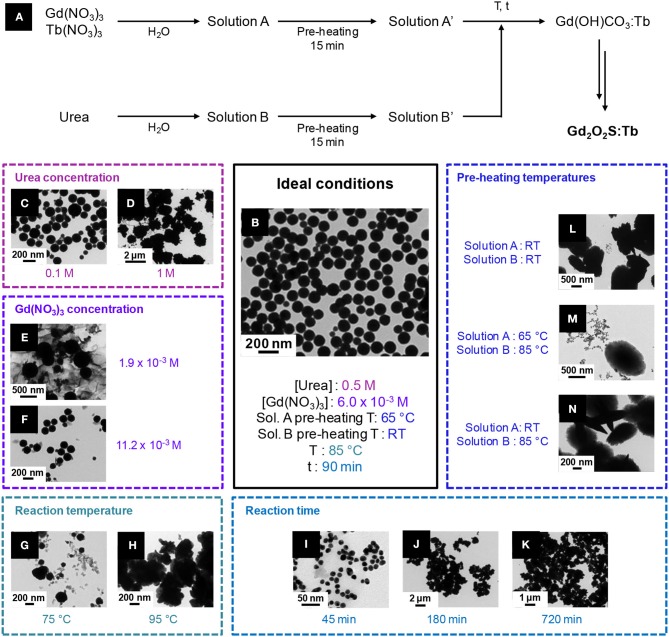
Optimized synthesis of Gd_2_O_2_S:Tb nanoparticles by Hernández-Adame et al. (2014) **(A)** Synthetic strategy: the authors first synthesized from nitrates and urea a doped hydroxycarbonate precursor that was later converted to the oxysulfide. TEM micrographs of the final Gd_2_O_2_S:Tb nanoparticles in the optimized conditions **(B)** and of the different Gd(OH)(CO_3_) particles obtained with through the optimization **(C–N)**. Only one parameter is changed at once, the others being identical to the optimal conditions. Adapted from Hernández-Adame et al. (2014) with permission of Elsevier.

In 2014, Hernández-Adame et al. extensively studied the influence of the reaction conditions on the morphology of Gd(OH)CO_3_:Tb and Gd_2_O_2_S:Tb, by mixing an urea aqueous solution with an aqueous solution of Tb(NO_3_)_3_ and Gd(NO_3_)_3_, and performing two thermal treatments (at 800°C under air and at 900°C under a N_2_/S atmosphere; Hernández-Adame et al., 2014). The precursor concentrations, the temperature of the stock solutions of nitrates and urea and the time and temperature of reactions were varied (Figure 12). Eventually, only one set of conditions gave regular spherical nanoparticles (Ø ≈ 100 nm): a nitrate solution at 6.0 10^−3^ M, pre-heated at 65°C, and a urea solution at 0.5 M, at room temperature, reacting for 90 min at 85°C (Figure 12B). Hernández-Adame et al. recently completed their work with a comprehensive study of the effects of the terbium concentration on the luminescence properties of their nanoparticles (Hernandez-Adame et al., 2018).

**Figure 13:**
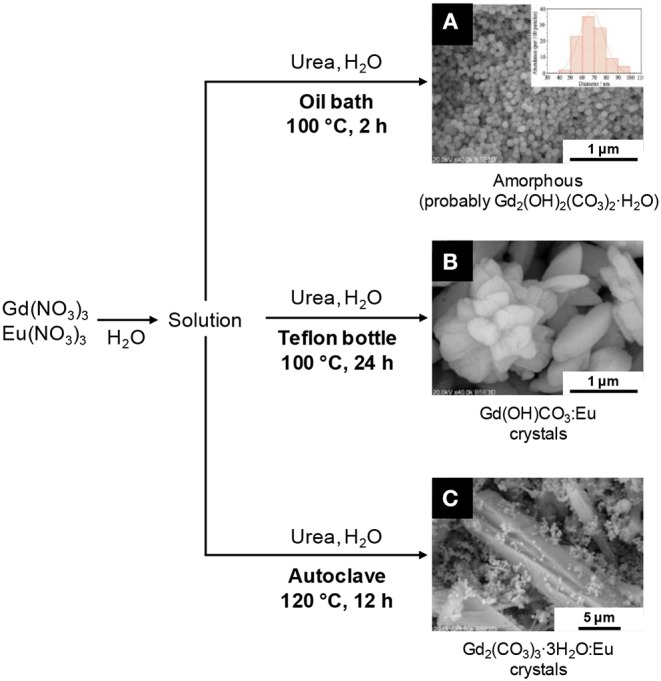
Structural and morphological variations of Gd_2_O_2_S precursors obtained by Cichos et al. (2016). Depending on the heating process, strong variations are observed: amorphous spherical nanoparticles (**A** with the size distribution in inset), hydroxycarbonate microcrystals **(B)** or carbonate microcrystals **(C)** can be obtained. Adapted from Cichos et al. (2016) with permission of Elsevier.

Recently, Cichos et al. studied three different syntheses of europium-doped Gd_2_O_2_S nanoparticles starting from nitrates and urea: (i) heating water at around 100°C for 2 h using an oil bath, (ii) heating a Teflon bottle at 100°C for 24 h, and (iii) heating an autoclave at 120°C for 12 h (see the autoclave section; Cichos et al., 2016). After reaction, the isolated solids were heated with an excess of sulfur under argon at 950°C for 1 h to yield Gd_2_O_2_S:Eu particles. In case (i), the intermediary solid was amorphous but the particles were spherical and quite monodisperse in diameter (Figure 13A). After sulfidation, crystalline Gd_2_O_2_S:Eu nanoparticles with a diameter close to 135 nm were obtained. The surface was rougher than the amorphous precursor's one. The Teflon bottle method [case (ii)] gave micrometric hydroxycarbonate Gd(OH)CO_3_ particles (Figure 13B) that were converted to Gd_2_O_2_S:Eu micrometric crystals and was thus not suitable for nanoparticles synthesis.

##### Other precipitation routes from aqueous solutions

Closely related to urea's precipitating method, an aqueous ammonia/ammonium hydrogenocarbonate precipitation of nitrates was reported by Tian et al. (2015). A NH_4_HCO_3_/NH_3_·H_2_O solution was added dropwise to a nitrate solution including Y(NO_3_)_3_, Yb(NO_3_)_3_, and Er(NO_3_)_3_. A white precipitate of Y(OH)_*x*_(CO_3_)_*y*_:Yb, Er was obtained and dried. The Y_2_O_2_S:Yb, Er nanoparticles were obtained using sulfur vapor (S_8_ heated at 400°C) carried by N_2_ at 900°C for 1 h. The small but aggregated crystalline nanoparticles (Ø ≈ 30 nm) were phase-pure, based on XRD. Here, the use of ammonium hydrogenocarbonate and aqueous ammonia enabled the authors to carry out the reaction without heating whereas urea needed thermolysis.

Regarding upconverting oxysulfide nanoparticles, Fu et al. (2010) chose Na_2_CO_3_ to form intermediate solids which were then sulfidated. After dissolution of Y(NO_3_)_3_, Yb(NO_3_)_3_, and Ho(NO_3_)_3_, the nitrate solution was added in a 0.1 M solution of Na_2_CO_3_ containing PEG 4000 as surfactant. A solid precipitated, was isolated and dried. It was heated at 600°C to yield Y_2_O_3_:Yb, Ho. Then, the oxide was converted to oxysulfide using Xing's thermal treatment described in the previous section. Interestingly, Na_2_CO_3_ enables the authors to work at ambient temperature in the first step whereas urea required thermolysis. However, two thermal treatments were necessary to reach the oxysulfide product. Moreover, an irregular faceted morphology and a significant polydispersity in size were found in the final sample.

#### Aqueous Reactions Under Autogenic Pressure

This section is dedicated synthesis in aqueous solution under pressure, in autoclave. We already mentioned the low boiling point of water as a strong limitation if we consider the temperatures commonly required for crystalline nanoparticles synthesis. Synthesis under pressure might be a way to overcome this limitation. Unfortunately, like the precipitation reactions at atmospheric pressure, the reported syntheses in hydrothermal conditions mainly focus on producing an intermediate solid that requires sulfidation in a second step (Table 4). Nevertheless, these syntheses expanded the range of available morphologies for the final oxysulfide nanoparticles.

**Table 4:** Hydrothermal syntheses of Ln_2_O_2_S nanomaterials.

**References** **Phase**	**Metal sources**	**Additives**	**Hydrothermal step**	**Annealing step**	**Morphology (size)**
Thirumalai et al. (2008b) Gd_2_O_2_S:Eu	Gd(NO_3_)_3_ Eu(NO_3_)_3_	NaOH (pH ≈ 11)	130–150°C 24–48 h	Ar/CS_2_/S/C, 700°C, 2–3 h	Nanotubes (50 nm × few μm)
(Thirumalai et al., 2009a)) Gd_2_O_2_S:Eu	Gd(NO_3_)_3_ Eu(NO_3_)_3_	NaOH (7 ≤ pH ≤ 13)	100–180°C 12–48 h	Ar/CS_2_/S/C, 700°C, 2–3 h	Tunable: Spheres (Ø 15 nm) + hexagonal crystals (20–30 nm) Nanosheets (15 ×80 nm^2^), Nanobelts (25 nm × few μm) Nanotubes (15 ×200 nm^2^), Nanorods (15 × 100 nm^2^) Nanowires (15 × 250 nm^2^)
Thirumalai et al. (2009b) Y_2_O_2_S:Eu	Y(NO_3_)_3_ Eu(NO_3_)_3_	NaOH (7 ≤ pH ≤ 13)	100–180°C 12–48 h	Ar/CS_2_/S/C, 600°C, 2 h	Tunable: Spherical (15 nm) + hexagonal crystals (20–40 nm) Nanosheets (15 × 70 nm^2^), Nanobelts (25 nm × few μm) Nanotubes (10 × 200 nm^2^), Nanorods (10 × 70 nm^2^) Nanowires (15 × 250 nm^2^)
Li et al. (2009) Y_2_O_2_S:Eu,Mg,Ti	Y(NO_3_)_3_	NH_3_·H_2_O	260°C 5 h	1/S_8_ in graphite (CS_2_), 800°C, 4 h 2/Eu_2_O_3_, TiO_2_, Mg(OH)_2_·4Mg(CO_3_)·6H_2_O, 1100°C, 4 h	Nanorods (50 × 400 nm^2^)
Li et al. (2010) Y_2_O_2_S:Eu,Mg,Ti	Y(NO_3_)_3_	NaOH (pH ≈ 14)	180°C 12 h	H_2_, S_8_, Na_2_CO_3_, Eu_2_O_3_, TiO_2_, Mg(OH)_2_·4Mg(CO_3_)·6H_2_O 600–800°C, 4 h	Hexagonal nanoparticles (30–50 nm)
Ai et al. (2010c) Y_2_O_2_S:Eu,Mg,Ti	Y(NO_3_)_3_	NaOH (pH ≈ 13)	180°C 12 h	1/S_8_ in graphite (CS_2_), 800°C, 4 h 2/Eu_2_O_3_, TiO_2_, Mg(OH)_2_·4Mg(CO_3_)·6H_2_O, 1100°C, 4 h	Nanotubes (100–200 nm × 1–3 μm)
Cui et al. (2014b), Liu et al. (2014b) Cui et al. (2014a); Liu et al. (2014a) Y_2_O_2_S:Eu,Mg,Ti	Y(NO_3_)_3_ Eu(NO_3_)_3_ Mg(NO_3_)_2_ Ti(OBu)_4_	NaOH (pH ≈ 13)	180°C 12 h	S_8_/C (CS_2_), 600–800°C, 6 h	Nanotubes (200 nm × 3 μm)
Huang et al. (2014) Y_2_O_2_S:Eu,Zn,Ti	Y(NO_3_)_3_ Eu(NO_3_)_3_ Zn(NO_3_)_2_ Ti(OBu)_4_	NaOH (pH ≈ 13)	180°C 12 h	S_8_/C (CS_2_), 600–800°C, 6 h	Nanotubes (200 nm × 3 μm)
Wang et al. (2015) Lu_2_O_2_S:Eu	Lu(NO3)3 Eu(NO3)3	NaOH (pH ≈ 11) Thiourea, PVP K30	200°C 24 h	S/N_2_, 600°C,2 h	Nanorods (20 × 500 nm^2^)
Cichos et al. (2016) Gd_2_O_2_S:Eu	Gd(NO_3_)_3_ Eu(NO_3_)_3_	Urea	120°C 12 h	S_8_, Ar, 950°C, 1 h	Irregular microcrystals (≥ 1 μm) + submicrospheres (300–500 nm)
Rosticher et al. (2016) Gd_2_O_2_S:Eu,Mg,Ti	Gd(NO_3_)_3_ Eu(NO_3_)_3_ Mg(NO_3_)_2_ TiCl_4_	NaOH (pH ≈ 8) Thioacetamide	200°C 2 h	Ar, 700°C, 2 h	Nanospheres (Ø = 20 nm) + facetted crystals (50–100 nm)
Yuan et al. (2016) Y_2_O_3_:Eu/Y_2_O_2_S:Eu	Y(NO_3_)_3_ Eu(NO_3_)_3_	NaOH	100°C 5 h	S_8_, N_2_, 600°C, 1 h	Irregular morphology (≤ 150 nm)

In the late 2000's, Thirumalai et al. (2008b, 2009a) reported the hydrothermal synthesis of Gd_2_O_2_S:Eu (Table 4, entries 1, 2). Starting with an amorphous precipitate (obtained by adjusting the pH of an aqueous solution of Gd(NO_3_)_3_ with NaOH), they obtained Gd(OH)_3_ nanoscaled materials (hexagonal nanocrystals, nanotubes, nanobelts, …) after the hydrothermal treatment. The influence of the pH of precipitation and the temperature and duration of the hydrothermal define the morphology of the material. After impregnation of the solid with Eu^3+^ ions in a aqueous solution, sulfidation was performed using a CS_2_ atmosphere generated by reaction of sulfur and carbon. The morphology of Gd(OH)_3_ was retained in the final Gd_2_O_2_S:Eu nanopowder, with only slight size decreases. The nanomaterials are well-crystallized and the morphology is finely adjustable varying the reaction conditions. Unfortunately, an undescribed sulfidation process is performed before the annealing step. It is probably similar to the one mentioned before for the composite hydroxide method conducted by the same group (Thirumalai et al., 2011a). Moroever, an original study on the photo-induced impedance is presented. Interestingly, the morphology is retained also with other lanthanides, as similar results were obtained by Thirumalai et al. (2009b) on Y_2_O_2_S:Eu (Table 4, entry 3).

The oxysulfides nanoparticles obtained by hydrothermal syntheses were also extensively studied by Li, Ai, Liu et al. who obtained Y_2_O_2_S:Eu,Mg,Ti nanoparticles (Table 4, entries 4, 5, and 6; Li et al., 2009, 2010; Ai et al., 2010c). This combination of doping ions is typical for persistent luminescence. Aqueous ammonia NH_3_·H_2_O was used as a base for precipitation of hydroxides. The authors then inserted the dopants by solid-solid reaction in the annealing step with Eu_2_O_3_, Mg(OH)_2_·4Mg(CO_3_)·6H_2_O, and TiO_2_. Moreover, they noticed that using CS_2_ formed *in situ*, rather than solid S_8_, is crucial to keep the morphology. With S_8_, the Y(OH)_3_ nanotubes turned into hexagonal nanoparticles after the annealing step.

The group of Cui and Liu also put great efforts on the characterization of such nanoparticles (Table 4, entries 7 and 8; Cui et al., 2014b; Huang et al., 2014; Liu et al., 2014a,c). Soluble sources [Eu(NO_3_)_3_, Mg(NO_3_)_2_ and Ti(OBu)_4_] were employed as reactants rather than solids for doping. Thus, a moderated sulfidation annealing temperature ( ≤ 800°C) was employed to yield Y_2_O_2_S:Eu, Mg, Ti nanotubes. The synthesis (Cui et al., 2014b), the influence of earth-alkaline or metal M^II^ ion (Huang et al., 2014; Liu et al., 2014b), the effect of the relative concentration of Mg^II^ and Ti^IV^ (Liu et al., 2014a), and the Eu^III^ concentration were separately studied (Cui et al., 2014a). Later, Yuan et al. (2016) also reported mild conditions to synthesize composite Y_2_O_3_:Eu/Y_2_O_2_S:Eu nanoparticles starting from soluble nitrate precursors (Table 4, entry 12; Yuan et al., 2016). The particles were crystalline but presented an irregular morphology. They were incorporated in dye-sensitized solar cells that were fabricated by the group. An enhancement of the cell efficiency was measured thanks to the light scattering properties of the nanocomposite.

A rare example of Lu_2_O_2_S:Eu nanocrystals was reported in 2015 by Wang et al. (Table 4, entry 9). PVP K30 followed by a solution of thiourea in ethanol were added to lutetium nitrate dissolved in a mixture of water and ethylene glycol. Perfectly regular nanorods were obtained after a thermal treatment with a sulfidizing atmosphere. Here again, the sulfidizing step mechanism was not studied in detail.

In 2016, Cichos et al. tested an hydrothermal synthesis (Table 4, entry 10) to yield doped Gd_2_(CO_3_)_3_:Eu particles in comparison with reactions at atmospheric pressure (Cichos et al., 2016). The authors noticed that this method is rather not adapted to the synthesis of nanoparticles: several populations are obtained, including micrometric irregular crystals (Figure 13C).

The synthesis reported by Rosticher et al. (2016) is a promising exception (Table 4, entry 11). The crucial difference lies in the sulfidation method. An excess of water-soluble thioacetamide was incorporated before the hydrothermal heating after precipitation of amorphous Gd(OH)_3_:Eu, Mg, Ti with NaOH. This allowed incorporation of sulfur before the annealing step, which could conveniently be performed under inert atmosphere. Its role was only to improve the cristallinity and the luminescence performances of the powder.

#### Conclusion

The formation of oxysulfide nanoparticles in water encounters several limits. Because of the aqueous solvent, excess oxygen favors the formation of intermediary phases such as hydroxides, hydroxycarbonates, or oxides. Only an adequate sulfidation annealing step at high temperatures enables the formation of the oxysulfide nanoparticles. Nevertheless, it can affect the morphology of the nanoparticles with aggregation and sintering.

Moreover, the synthesis of the intermediary phases is also challenging. Precise reaction parameters have to be employed, with long optimization processes. In Figures 12, 13, we reminded for instance the works of Hernández-Adame et al. and Cichos et al. on the synthesis of doped gadolinium oxysulfide nanoparticles with urea. Not only the reaction temperature and time had a great effect on the final morphology of the intermediates: concentrations of the reactants, pre-heating temperatures, heating techniques are also crucial to obtain the desired product.

Working in organic medium then seems to be a suitable solution to overcome the excess available oxygen.

### Syntheses in Organic Medium

The following section is dedicated to the reactions mainly performed in organic medium. Thanks to the availability of high boiling-point solvents, the temperature of the reaction medium can reach 200-300°C much easier than in water. Moreover, the control of the nanoparticles size and morphology in organic solvents is easily attainable using surfactants.

**Scheme 4:**
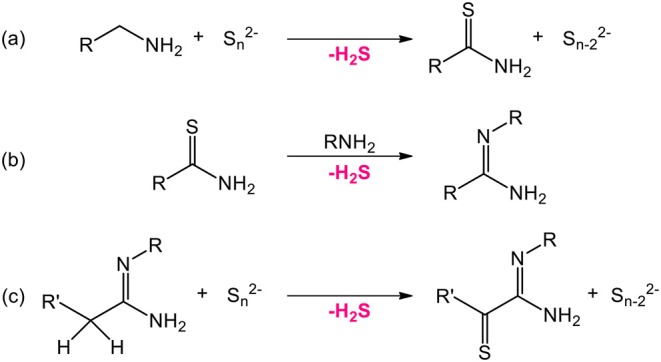
Reaction pathways releasing H_2_S in a primary amine/elemental sulfur medium at 130°C as proposed by Thomson et al. (2011) Water traces can also react with the thioamide to form the corresponding amide and H_2_S similarly to reaction (b).

In the case of lanthanide oxysulfide nanoparticles, we also note several benefits of organic medium for the stoichiometry:

- the control of the oxygen concentration that is assured by the absence of excess reactive oxygen brought by water and reactions under inert atmosphere,
- the use of molecular sulfur sources with for instance the possibility of decomposing hydrophobic single-source precursors (typically, lanthanide complexes with sulfur-containing ligands) or the dissolution and activation of elemental sulfur in primary amines, as they react to form reactive alkylammonium polysulfides which release *in situ* H_2_S (Scheme 4; Thomson et al., 2011).

In both cases, the amount of reactive anions can be set to the desired value by playing on the concentration and the nature of the reactants. In water-based reactions, an excess of water in the precipitation step was followed by an excess of sulfur during the annealing step. Thus, organic medium brings the possibility to finely control the stoichiometry of the anions and one could expect that it leads to different oxysulfide compositions apart from thermodynamics considerations.

#### Decomposition of Sulfur-Containing Single-Source Precursors

**Scheme 5:**
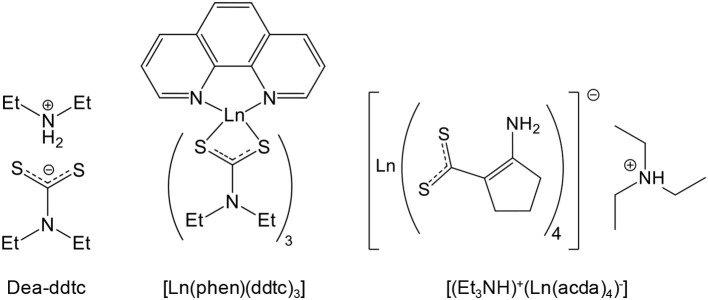
Chemical formulas of diethylammonium diethyldithiocarbamate (dea-ddtc), [Ln(1,10-phenanthroline)(diethyldithiocarbamate)_3_] complex ([Ln(phen)(ddtc)_3_]), and Triethylammonium of tetra(2-aminocyclopentenedithiocarbamate) lanthanide ([(Et_3_NH)^+^(Ln(acda)_4_)^−^]).

**Figure 14:**
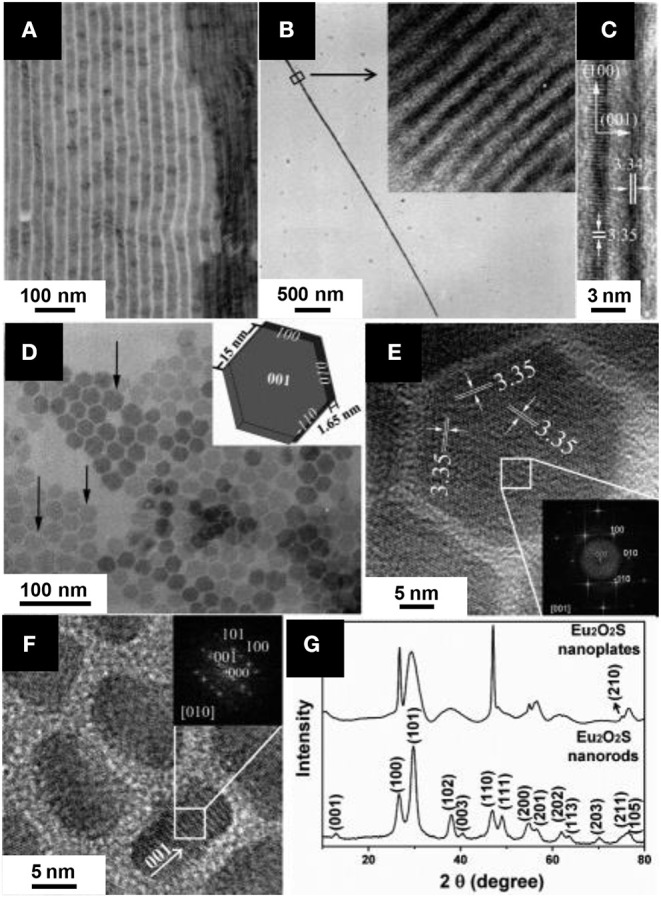
Characterization of Eu_2_O_2_S nanoparticles obtained by decomposition of [Eu(phen)(ddtc)_3_] by Zhao et al. (2006a) TEM micrographs of self-assemblies formed by the hexagonal nanoplates **(A)**, of a single nanowire **(B)** with a zoom in inset and HRTEM micrograph of two nanowires **(C)**. **(D)** TEM micrographs of hexagonal nanoplates lying on their flat surface. Inset: representation of a single nanoplate. HRTEM images of Eu_2_O_2_S nanoplates lying on their flat surface **(E)** and of short nanorods **(F)**. Insets are FFT of the indicated regions. **(G)** Powder XRD pattern of the Eu_2_O_2_S nanoparticles. Adapted with permission from Zhao et al. (2006a), copyright (2006) American Chemical Society.

The decomposition of lanthanide complexes bearing ligands with sulfur in the presence of dioxygen can lead to oxysulfide nanoparticles. It was shown for the first time in 2006 in a communication by Zhao et al. who developed the synthesis of thin monodisperse hexagonal nanoplates of Eu_2_O_2_S, Sm_2_O_2_S and Gd_2_O_2_S (Zhao et al., 2006a). In a mixture of organic solvents and surfactants typical for colloidal synthesis (1-octadecene, oleic acid and oleylamine), [Eu(phen)(ddtc)_3_] (phen = 1,10-phenanthroline, ddtc = diethyldithiocarbamate; Scheme 5) was decomposed under air at 290°C in 45 min, forming anistropic nanocrystals (15 × 1.7 nm (Flahaut et al., 1958; Figure 14). For the first time, the observation of self-assembled oxysulfide nanoplates to nanowires is made (Figures 14A–C). The nanoplates are piled one above each other, because of the hydrophobic interaction between the surface surfactant chains of oleic acid (oleylamine-metal bonds are weaker than oleic acid-metal bonds; Cheon et al., 2004).

Interestingly, EuS (Eu^II^) nanocrystals were obtained with the same synthesis but under inert atmosphere with oleylamine alone (which played the role of reducing agent; Zhao et al., 2006b). A more detailed study on the pyrolysis of the [Ln(phen)(ddtc)_3_] precursor and the nanoparticles properties was also reported. A noticeable work using the same strategy was conducted by Tan et al. (2016). In comparison with europium, the decomposition of [La(phen)(ddtc)_3_] and [Pr(phen)(ddtc)_3_] only yielded LaS and PrS. From oxidation of the sulfides, oxysulfates nanoparticles of La_2_O_2_SO_4_ and Pr_2_O_2_SO_4_ were obtained. The nanoparticles of Eu_2_O_2_S, La_2_O_2_SO_4_, and Pr_2_O_2_SO_4_ were then tested for the water-gas-shift reaction.

Lin et al. (2016) obtained europium- and terbium-doped Gd_2_O_2_S and europium-doped Tb_2_O_2_S by decomposition of the same precursor. Although the morphology of the nanoparticles was not perfectly regular, and the crystallinity not optimal, an extensive luminescence study was performed and biologic tests (*in vivo* imaging, cell viability) were conducted. The latter required a coating with 3-aminopropyltriethoxysilane (Figure 10) and grafting of methoxy-polyethyleneglycol and Alexa Fluor 660 (photostable red dye which emits photons in the wavelength range of 630-650 nm).

In 2012, He et al. (2009) described a similar decomposition of a precursor formed *in situ*. The reaction yielded europium oxysulfide nanorods. In this synthesis, europium oleate, oleylamine, 1,10-phenanthroline, and dodecanethiol were heated at 320°C under inert atmosphere before hot injection of diethylammonium diethyldithiocarbamate (dea-ddtc, Scheme 5) dissolved in oleylamine. Nanorods were isolated after 1 h of reaction. The oxygen source was not explicitly discussed, but it was likely the oleate ions in the europium-oleate complex. Even if dea-ddtc is the most probable sulfur source, the introduction of dodecanethiol was not discussed. Nevertheless, this report showed that forming the single-source precursor *in situ* was a viable strategy. The non-stoichiometric character of the Eu_2+x_O_2_S nanoparticles was evidenced by the Eu/S ratio measured by EDS. Non-stoichiometry is attributed to Eu^II^ in the solid and was already observed for the bulk phase in the 1960's by Ballestracci and Quezel who estimated that 1% of the europium atoms were divalent thanks to neutron diffraction and magnetic measurements (Ballestracci et al., 1968; Quezel et al., 1970). He et al. (2009) also described magnetic properties of europium oxysulfide nanoparticles and confirm the Eu^II^/Eu^III^ ratio, even if the average composition Eu_2.11_O_2_S corresponds to about 15% of Eu^II^. Moreover, an electrophoretic deposition of the nanorods was proposed.

**Scheme 6:**
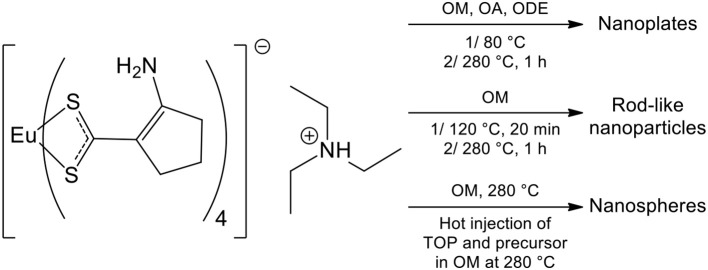
Triethylammonium of tetra(2-aminocyclopentenedithiocarbamate) europium ([(Et_3_NH)^+^(Eu(acda)_4_)^−^]) decomposition in organic solvents by (Ghosh et al., 2016)

Ghosh et al. reported another precursor to obtain Eu_2_O_2_S nanoparticles (Ghosh et al., 2016). According to the authors, La_2_O_2_S and Nd_2_O_2_S can also be prepared with a similar procedure. Synthesized from europium nitrate, triethylamine (Et_3_N) and 2-aminocyclopentene-1-dithiocarboxylic acid (Hacda), [(Et_3_NH)^+^(Eu(acda)_4_)^−^] was decomposed via three different methods (Scheme 6).

By heating the precursor in an OM/OA/ODE mixture, ultrathin nanoplates of Eu_2_O_2_S were obtained. However, the 0.3 nm reported thickness is quite surprising, as it would represent a single monolayer of the solid, and there is no correlated peak extinction in the corresponding XRD pattern. Using similar conditions with OM only led to rod-like nanoparticles (7 × 3 nm^2^). Finally, hot injection of [(Et_3_NH)^+^(Eu(acda)_4_)^−^] and trioctylphosphine (TOP) led to polydisperse nanospheres with an average diameter of 13 nm. The catalytic activity of Eu_2_O_2_S, and especially its activity as a peroxidase mimic, was deeply investigated. Because Eu_2_O_2_S catalyzed the oxidation of 3,3′,5,5′-tetramethylbenzidine (TMB) in presence of H_2_O_2_ and neither La_2_O_2_S nor Nd_2_O_2_S succeeded in it, the authors concluded to a mechanism involving the Eu^III^/Eu^II^ redox couple.

#### Syntheses With High Boiling-Point Organic Solvents at Atmospheric Pressure

**Figure 15:**
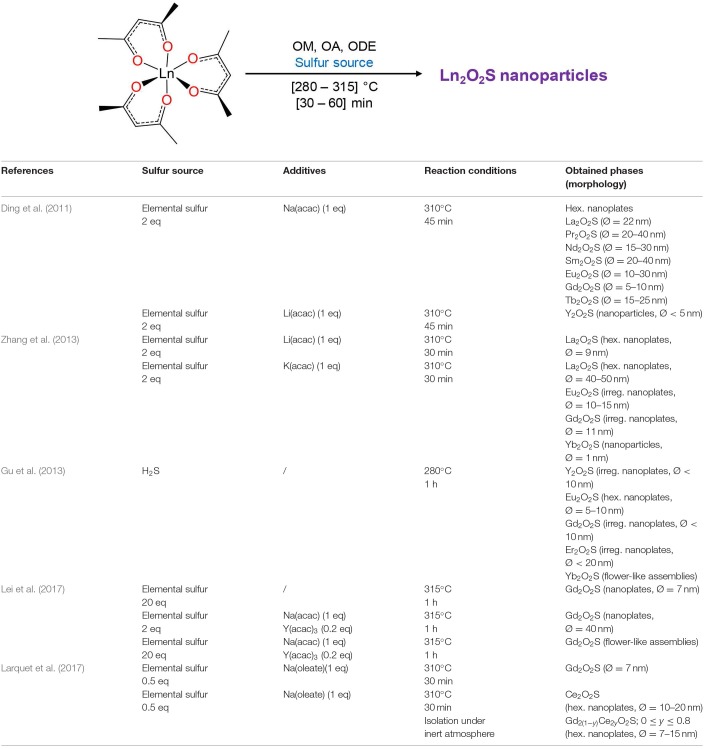
Ding's alkali-based synthesis of lanthanide oxysulfide and its derivatives. The number of equivalent (“eq” in the table) is the molar ratio between the reactant and metal. The term “hex.” stands for “hexagonal” and “irreg.” for “irregular.”

Also colloidal synthesis in organic solvents have been used for years in the synthesis of metal and metal oxide nanoparticles, the first report for metal oxysulfides was published by Ding et al. (2011). Lanthanide acetylacetonate Ln(acac)_3_ (1 equiv.), elemental sulfur (1 equiv.), and sodium acetylacetonate (1 equiv.) were added in an OM/OA/ODE mixture and heated for 45 min. at 310°C under inert atmosphere after degassing under vacuum at 120°C (Figure 15). Size-monodisperse hexagonal nanoplates of Ln_2_O_2_S were obtained. They were thin (a few monolayers) and 5–40 nm wide depending on the lanthanide. The composition of the powder showed a lack of sulfur (Na_0.4_La_1.6_O_2_S_0.6_), which was attributed to terminal [Ln_2_O_2_]^2+^ layers. The crucial advantage of this method is its high versatility: La_2_O_2_S, Pr_2_O_2_S, Nd_2_O_2_S, Sm_2_O_2_S, Eu_2_O_2_S, Gd_2_O_2_S, Tb_2_O_2_S were prepared. The sodium ions, added in stoichiometric amounts, were proposed to help the crystallization and favor the oxysulfide formation. The hypothesis of the authors is that the close ionic radii of sodium [*r*(Na^I^(VII)) = 1.26 Å] and larger lanthanide ions [*r*(La^III^(VII)) = 1.24 Å to *r*(Tb^III^(VII)) = 1.12 Å] enables cation exchanges in the solid and favors the oxysulfide crystallization. Lithium ions were tested and were efficient for Y_2_O_2_S synthesis. In 2013, a more complete study (experimental study and calculations based on density functional theory) on the alkaline additives on the formation and morphology of the obtained nanocrystals also showed the possible use of potassium to synthesize oxysulfide nanoparticles (La_2_O_2_S, Eu_2_O_2_S, Gd_2_O_2_S, and Yb_2_O_2_S; Zhang et al., 2013). The hypothesis of alkali insertion in the crystal structure was recently disputed: the alkali would serve as a stabilizing species for the formation of a lamellar alkali-oleate phase (as observed with sodium) rather than as a doping ion in the Ln_2_O_2_S structure (Larquet et al., 2020). In 2017, Lei et al. investigated the roles of yttrium and sodium in the formation and growth of Gd_2_O_2_S, by using them separately or combined. They also demonstrated that a large excess of sulfur allows forming gadolinium oxysulfide nanoplates without adding sodium ions (Lei et al., 2017).

In 2017, Tan and Li announced the formation iron/sodium co-doped lanthanum oxysulfide nanoparticles (Na, La)_2_O_2_S:Fe (Tan and Li, 2017). Such doping with light transition metal is very rare due to ionic radii mismatch. Thus, according to the authors, only a limited amount of iron would have been able to substitute lanthanum, and surprisingly, no contraction of the lattice was observed despite the ionic radii difference [*r*(La^III^(VII)) = 1.24 Å; *r*(Fe^III^(VII)) ≈ 0.85 Å]. Even though such iron doping would be very interesting for catalytic features, it is quite unclear that iron was well-inserted in the La_2_O_2_S phase. In 2015, Jiang et al. employed Ding's synthesis and demonstrated the possible use of La_2_O_2_S:Eu nanoparticles as optical temperature sensors (“nanothermometer”) (Jiang et al., 2015). Our group recently investigated the reactivity of Ln_2_O_2_S hexagonal nanoplates formed with a stoichiometric amount of sulfur and demonstrated different oxidation processes in bimetallic Gd_2(1−y)_Ce_2y_O_2_S nanoparticles. It enabled us to prove that highly unstable Ce_2_O_2_S nanoparticles can also be formed with the help of sodium ions, as long as it is isolated and stored under strict inert conditions (Larquet et al., 2017). The thermal stability of the nanoparticles was investigated under inert and oxidizing atmosphere, highlighting the possibility to remove the oleate surface ligands by a mild thermal treatment (Larquet et al., 2019a). Moreover, both magnetic properties (Larquet et al., 2019b) and optical ones (bandgap) (Larquet et al., 2019c) could be tuned as a function of the Gd:Ce ratio.

Gu et al. managed to obtain yttrium, gadolinium, erbium, and ytterbium oxysulfide nanoplates using oleylamine as only solvent and H_2_S as sulfurating agent. Ln(acac)_3_ and oleylamine were degassed at 120°C and then heated at 280°C for 1 h under a H_2_S/N_2_ flow (20/80 *v*/*v*, 60 mL/min) to yield Ln_2_O_2_S nanoplates (Gu et al., 2013). Again, sodium ions were shown to help the crystallization of the nanoplates but were not necessary in this case. Y_2_O_2_S, Eu_2_O_2_S, Gd_2_O_2_S, Er_2_O_2_S, and Yb_2_O_2_S were prepared by this route.

**Figure 16:**
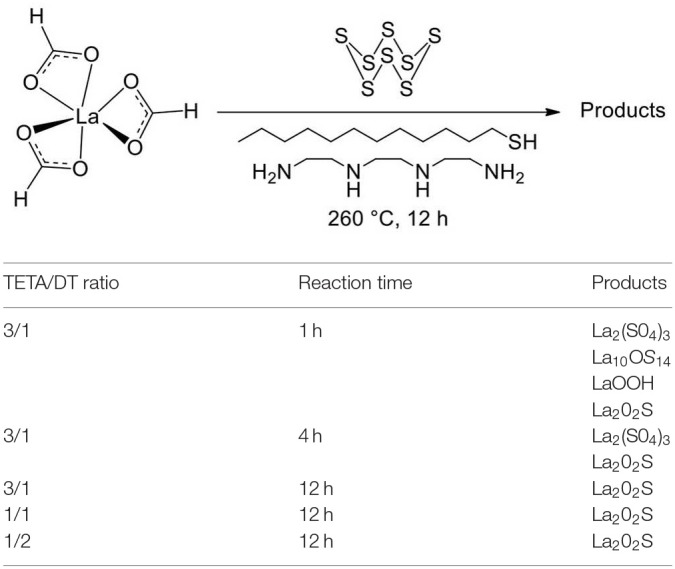
Reaction of Ma et al. (2013) to yield La_2_O_2_S nanoparticles.

Another method was reported in 2013 by Ma et al. (2013) to synthesize europium-doped lanthanum oxysulfide La_2_O_2_S:Eu. Lanthanide formates La(HCOO)_3_ and Eu(HCOO)_3_ were heated at 260°C in the presence of elemental sulfur (2 equiv. of S) in triethylenetetramine (TETA) and dodecanethiol (DT) (Figure 16). After 12 h, La_2_O_2_S:Eu nanocrystals were obtained with triethylenetetramine/dodecanethiol ratio being 1:2 and the nanospheres diameter was around 100 nm. Without dodecanethiol, 100 nm in width and 10 nm in thickness La_2_O_2_S:Eu nanoplates were obtained.

The amine/thiol ratio influenced the morphology. When the TETA/DT ratio was 3:1 or 1:1, micronic structures were obtained. Interestingly, other precursors [La(NO_3_)_3_, LaCl_3_, La_2_O_3_, and La(OH)_3_] were not selective enough or did not completely react [impurities of La(OH)_3_ or La_2_O_3_ based on XRD]. Also, shorter reaction times with TETA/DT = 3:1 for the exhibited the rare La_10_OS_14_ intermediary phase with LaOOH and La_2_(SO_4_)_3_. Although the crystals are large and the selectivity can be improved, it is to the best of our knowledge the only occurrence of a promising protocol for nanoscaled Ln_10_OS_14_.

#### Solvothermal Syntheses in Autoclave

In 2000, Li et al. tested a direct and simple solvothermal sulfidation process for numerous lanthanide oxides. Ln_*x*_O_*y*_ powders (Ln = Y, Sc, La, Pr, Nd, Eu, Sm, Gd, Ho, Er, Yb, or Lu) and S_8_ were suspended in ethylenediamine and heated in autoclave at 150°C for 8 h (Li et al., 2000). Aggregated and irregularly-shaped crystalline spherical nanoparticles of Pr_2_O_2_S, Eu_2_O_2_S, and Gd_2_O_2_S were obtained with this method (<50 nm). The authors suggested an anion-exchange mechanism between the O^−II^ in the Ln_*x*_O_*y*_ crystal and the S^−II^ available in polyanions when S_8_ reacts with TETA. They supposed next that oxysulfide nuclei could leave the surface of the oxide to grow apart.

For Ln = La, Nd, Sm, Ho, and Er, the conversion was incomplete (Ln_2_O_2_S and Ln_*x*_O_*y*_ on the XRD pattern) and no change was observed with longer reaction times. Only the starting oxide was observed for Ln = Y, Sc, Yb, and Lu. In 2012, Gd_2_O_2_S:Eu and Gd_2_O_2_S:Er, Yb nanoplates were also obtained by Liu et al. in ethylenediamine using gadolinium nitrate and elemental sulfur (Liu et al., 2012). PVP (K29-32) or OM was added to a solution of the lanthanide nitrate in ethanol. The resulting solution was added dropwise into ethylenediamine and sulfur. The autoclave was heated at 220°C for at least 4 h to form crystalline hexagonal nanoplates. With OM, subsequent aggregation in flower-like structures was observed. Separated nanocrystals of irregular shape were obtained with PVP. Y_2_O_2_S:Eu and Y_2_O_2_S:Er, Yb were obtained from yttrium acetate by the same group with PVP and a thermal treatment at 250°C for 24 h (Liu et al., 2014a). Various self-assemblies of the nanoparticles were formed, depending on the presence of PVP, sulfur concentration, and so on.

Song et al. (2010) obtained Gd_2_O_2_S:Eu and Gd_2_O_2_S:Tb nanospheres from the solvothermal treatment of lanthanide nitrates in a mixture of ethanol and ethylene glycol, containing polyvinylpyrrolidone (PVP K30, M = 40,000 g/mol) and thiourea. The autoclave was heated at 200°C for 24 h and the isolated solid was then sulfidized in a N_2_/S atmosphere at 600-800°C to form doped gadolinium oxysulfide nanoparticles. PVP is believed to be responsible for the spherical morphology, and polymer residues were evidenced on the rough surface of the nanoparticles. They presented a good crystallinity and a good monodispersity in diameter. Their size was tunable between 150 nm and 1.25 μm by varying the PVP content and the ethanol/ethylene glycol ratio. A similar strategy was used by Deng et al. to yield Y_2_O_2_S:Sm hollow nanospheres (Ø = 140-200 nm; Deng et al., 2012a). The thermal treatment was based on Li's work (Y_2_O_2_S nanoparticles hydrothermal synthesis, Table 4, entry 4; Li et al., 2009). The authors first proposed a mechanism involving H_2_S/CO_2_ bubbles to explain the holes, but finally declared in a second paper on Y_2_O_2_S:Eu, Mg, Ti nanoparticles that NH_3_/CO_2_ bubbles were more likely the templating agents (Deng et al., 2012b). Similarly to Song's spheres, the surface was rough and the nanoparticles seemed to be constituted with smaller units.

Thirumalai et al. (2011b) have mainly focused their work on water-based syntheses, but also prepared various morphologies of Gd_2_O_2_S:Eu nanoparticles in oleylamine. GdCl_3_·6H_2_O and EuCl_3_·6H_2_O were introduced in hot oleylamine and various amounts of thioacetamide were added. The resulting solution was heated in an autoclave at 120-240°C for 12-24 h. Flower-like nanocrystals (≈10 nm), nanospheres (Ø = 5-10 nm) and nanorods of various lengths (Ø = 6 nm) were obtained depending on the reaction conditions and the thioacetamide amount. An excess of sulfur was proposed to be mandatory to ensure a high chemical potential, which promoted the formation of nanorods. Despite the good morphology control, the XRD patterns of the nanoparticles showed a poor crystallinity of the materials: only broad peaks were observed. It is intriguing because nanorods presented big crystal domains, and HRTEM images showed large and regular lattices.

## Transition Metal Oxysulfides Nanoparticles

Transition metal bulk oxysulfides are quite rare. Zinc, titanium, molybdenum, and tungsten oxysulfide were nevertheless obtained. Most of the time, they were obtained under the form of amorphous thin films or particles.

Because of the electronegativity and atomic number differences between the two anions, transition metals will preferentially bind to one of them (in the hard and soft acids and bases theory, oxygen is a hard base and sulfur a soft base). Also, keeping reduced sulfur *id est* avoiding sulfates or other oxidized sulfur species is highly difficult because of their good thermodynamic stability.

### Challenging Synthesis, Tricky Characterization

In the previous section, we detailed numerous syntheses of Ln_2_O_2_S nanoparticles. It is an exception in the oxysulfide family, as it remains to the best of our knowledge the only structure for which monophasic crystalline nanoparticles could be formed. Today, a vast and promising land of metal oxysulfide nanoparticles, especially involving transition metals, must be explored. With such nanoparticles with transitions metals and chalcogens, new applications could emerge, such as heterogeneous catalysis, photocatalysis, battery materials, superconduction, and so on.

Several advantages are intrinsically brought by soft reaction conditions (compared with typical synthesis of bulk crystals) and the nanoscale. Mild temperatures and small grain size can unlock metastable structures. Also, diffusion processes are much faster over nanometric distances and lead to efficient substitution reactions with nanoscaled materials. It opens new synthetic strategies to transform preformed oxide, sulfide, or metal nanoparticles in oxysulfide nanoparticles.

However, synthesizing transition metal ternary oxysulfides is particularly challenging. The ionic radius difference between O^−II^ (1.26 Å) and S^−II^ (1.70 Å) associated with the variable affinities with the metal make the substitution reactions highly difficult. Energy input by heating is especially not recommended for nanoparticles synthesis because of excessive growth and sintering.

Despite these difficulties, transition metal oxysulfide nanoparticles were already prepared. A few examples will be detailed in the next section. The main issue consists of identifying and evidencing the oxysulfide nature of the compound. Because excessive heating tends to stabilize sulfate or separate oxides and sulfides rather than crystallize an oxysulfide structure, the reported structures are mainly amorphous.

Identification and characterization of such phases is much harder than crystalline nanoparticles. In particular, inductively coupled plasma atomic emission spectroscopy (ICP-AES), X-ray fluorescence (XRF), and energy dispersive X-ray spectroscopy (EDS) are suitable techniques to evidence the presence of sulfur, but the determination of the nanoparticles' precise oxygen content remains a challenge. High resolution transmission electron microscopy and energy filtered transmission electron microscopy (EFTEM) constitute an elegant solution, but requires well-dispersed nanoparticles and will not provide accurate quantitative data. Moreover, it is hard to conclude about the precise localization of the atoms: are they in the whole particle or only at the surface (because of ligands for instance)?

The identification of the nature of the chemical bonds and oxidation states inside the material is a supplementary issue, yet this is required to differentiate oxysulfides from sulfates. It becomes highly problematic when the composition of a solid is unclear. Infrared and Raman spectroscopies are particularly appropriate for amorphous oxysulfide identification because M-O and M-S bonds generally present very distinguishable signatures. However, only qualitative analysis is possible. X-Ray photoemission spectroscopy (XPS) brings some clues but investigates only the very surface. X-ray absorption spectroscopy, such as XANES or EXAFS (at O K-edge, S K-edge, M K, L, or M-edge) is able to characterize the whole sample and gives precious information on the oxidation states and chemical bonds. However, surface and core cannot be distinguished and only average information is obtained, so that one should be very careful about hypothesis and interpretations. Furthermore, it must be noticed that the required energies for the different edges involves the use of different X-ray ranges (soft for oxygen, tender for sulfur, hard for the metal K-edge) and consequently the use of distinct beamlines. The analysis of the pair distribution function of the diffuse background of X-ray diffraction patterns (PDF) is expected to bring solutions as it can be applied to the analysis of amorphous compounds. Still, it remains a poorly studied technique in the field of nanoparticles analysis.

Finally, we emphasized the fact that oxysulfide nanoparticles can be metastable or unstable phases. It reinforces the difficulty to store, transfer, manipulate, and characterize them (for instance in air-filled room atmosphere and devices or when heating upon irradiation by electron or X-ray beams).

### Amorphous and Crystalline Cobalt Oxysulfide

**Figure 17:**
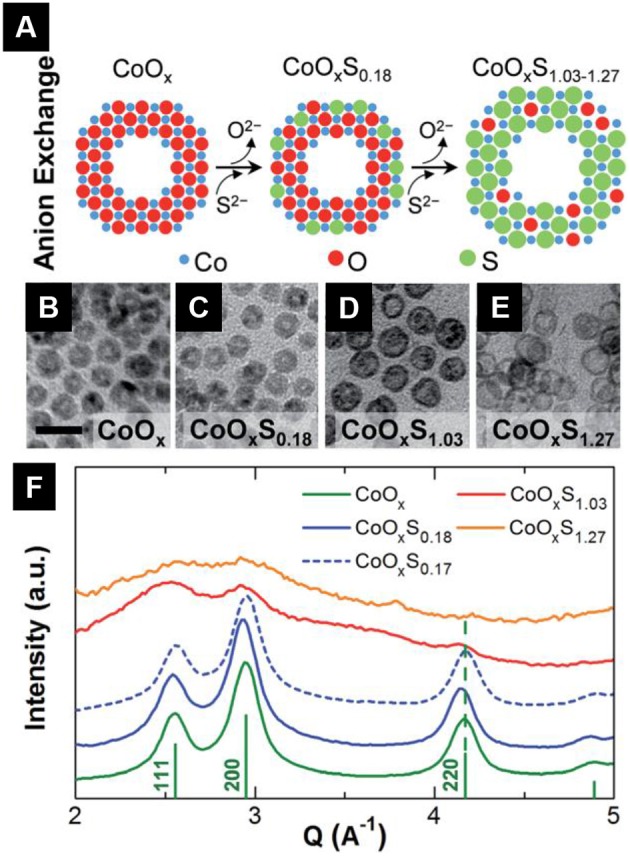
Adapted from Nelson et al. (2016) **(A)** Synthetic strategy of CoO_*x*_S_*y*_ hollow nanoparticles. TEM micrographs of CoO_*x*_S_*y*_ nanoparticles with *y* = 0 **(B)**; 0.18 **(C)**; 1.03 **(D)**; 1.27 **(E)**. **(F)** Rotationally averaged SAED patterns of the various CoO_*x*_S_*y*_ nanoparticles. Reference lines in green indicate CoO Bragg peaks (JCPDS 00-048-1719). Adapted from Nelson et al. (2016) with permission of the Royal Chemical Society.

In 2016, Nelson et al. reported the formation of cobalt oxysulfide CoO_*x*_S_*y*_ hollow nanoparticles (Nelson et al., 2016). The strategy consisted in the substitution of oxygen anions by sulfur anions in cobalt oxide hollow nanoparticles, using ammonium sulfide dissolved in oleylamine at 100°C (Figure 17A). The sulfur content was adjustable via the nominal (NH_4_)_2_S amount, with a saturation of the sulfur content at *y* ≈ 1.3. With low sulfur contents (*y* < 0.2), the particles keep the crystalline structure of CoO but with higher sulfur contents, the nanoparticles became amorphous (Figure 17F). The hollow nanosphere morphology was preserved during the whole experiment (Figures 17B–E).

No direct proof of the oxidation state of sulfur is brought by the authors. Nevertheless, annealing the sulfur-rich nanoparticles led to the formation of cobalt sulfides (possibly in a mixture with CoO). It supported the presence of reduced sulfur in the nanoparticles.

### Crystalline ZnO_1-*x*_S*_*x*_* Nanoparticles

**Figure 18:**
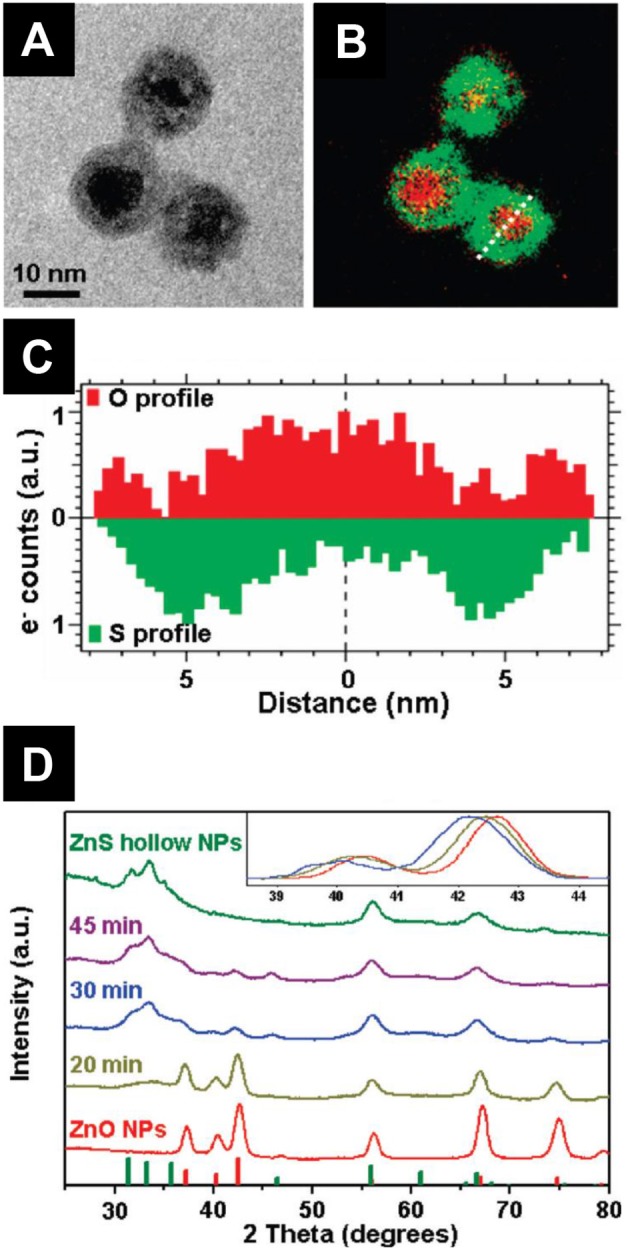
TEM **(A)** and EFTEM **(B)** images of ZnO@ZnS nanoparticles. **(C)** Oxygen and sulfur composition along the cross-section in **(B)**. **(D)** XRD patterns of the nanoparticles from ZnO to ZnS through ZnO@ZnS core-shell nanoparticles. Inset: Normalized pattern in the [38.5°; 44.5°] 2θ region. The small shifts of the diffraction peaks toward low 2θ values indicate a lattice dilatation caused by sulfur insertion. Adapted with permission from Park et al. (2009), copyright (2006) American Chemical Society.

Crystalline zinc oxysulfide was obtained at the nanoscale. In 2009, Park et al. carried out the substitution of oxygen atoms in ZnO by sulfur using hexamethyldisilathiane (Scheme 1) and obtained ZnS crystalline hollow nanoparticles (Figure 18; Park et al., 2009). The driving force of the reaction with ZnO is the formation of very stable Si–O bonds.

During the substitution process, the authors were able to isolate ZnO@ZnS core-shell crystalline nanoparticles, which are composed by a core of ZnO and a shell of isostructural ZnS wurtzite structure.

**Figure 19:**
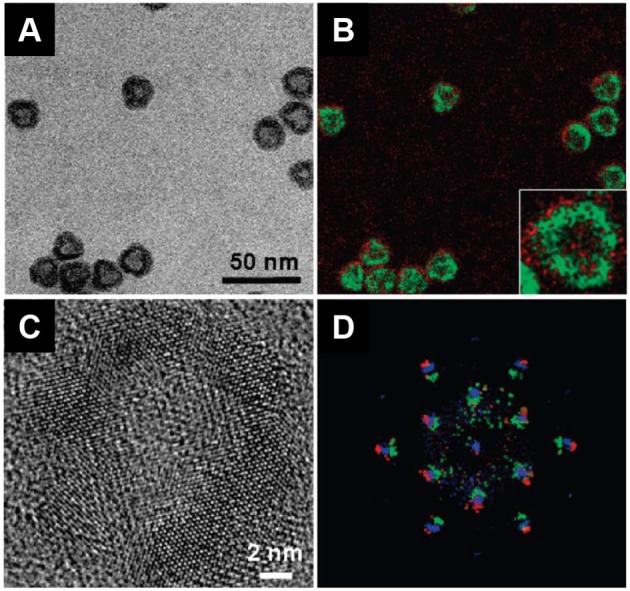
TEM **(A)**, EFTEM **(B)**, and HRTEM **(C)** of annealed core-shell nanoparticles. EFTEM strongly suggest the formation of a ZnO_1−x_S_*x*_ nanoalloy. **(D)** FFT image of **(C)** overlaid on FFT image of the precursor ZnO@ZnS core-shell nanoparticles (red: ZnO; green: ZnS; blue: annealed nanoparticles). These images support the heteroepitaxial formation of the ZnS shell and ZnO_1−x_S_*x*_ when thermally annealed. Adapted with permission from Park et al. (2009), copyright (2006) American Chemical Society.

The process was accompanied by the so-called “nanoscale Kirkendall effect,” which refers to the hollowing of the nanoparticles as a consequence of unbalanced diffusion rates (Wang et al., 2013). Because Zn^II^ diffuse outwards faster than S^−II^ inwards, the reaction finally led to a hollow ZnS structure. HRTEM and EFTEM also showed that the final ZnS nanoparticles were obtained through the formation of heteroepitaxial ZnO@ZnS intermediates that release the high interface energy by the diffusion of the core into the shell (Figure 19). The composition analyses of core-shell intermediates indeed showed that oxygen is not only localized in the core of the nanoparticle, but also in the shell. It suggested that the substitution process with hexamethyldisilathiane took place in the shell region where oxygen had migrated.

The reaction resulted in the formation of crystalline ZnO_1−x_S_*x*_ located in the shell. Furthermore, the authors were able to obtain pure hollow ZnO_1−x_S_*x*_ nanoparticles by thermally annealed the core-shell intermediates. Interestingly, the diffusion processes spontaneously occurred without sulfurating reagents and led to hollow ZnO_1−x_S_*x*_ alloys.

The research on zinc oxysulfide nanoparticles has grown in the last years. Pandey et al. (2013, 2014) managed to obtain the whole composition range (0 ≤ *x* ≤ 1) of nanoparticles. They obtained ZnO_1−x_S_*x*_ crystalline nanoparticles by a solution combustion method. Zn(acetate)_2_ and thiourea were incorporated in a mixture of ethanol and ethylene glycol (4:1) and then placed in a hot furnace (350°C) for 2 h. They showed that the bandgap varied with the sulfur content and that ZnO_1−x_S_*x*_ nanoparticles can photocatalyze the degradation of methyl orange. In 2017, Zhang et al. underlined the importance of doped zinc oxide to understand the intriguing ferromagnetic properties of certain *d*^*0*^ components and used the same combustion method than Pandey (Zhang et al., 2017). As a consequence of oxidation by air, they measured a significant amount of sulfate groups at the surface of their nanoparticles using XPS. Abdullah et al. (2017) synthesized ZnO_1−x_S_*x*_ nanoparticles using zinc(II) acetate and thioacetamide for the photocatalysis of the hydrogen evolution reaction. Gultom et al. (2017) in the same group also showed that nickel-doped ZnO_1−x_S_*x*_ nanoparticles were suitable for hydrogen production.

### Other Proposed Transition Metal Oxysulfides

Crystalline nano-aggregates of cobalt nickel oxysulfides (CoNi)O_*y*_S_*z*_ were claimed by Liu (2013). However, the author noticed that sulfur only existed in oxidized species (S^IV^, S^VI^) in the material using XPS. By definition, it cannot be called “oxysulfide” but should rather be named “oxysulfate.”

In 2017, Liu et al. reported “fullerene-like oxysulfide hollow nanospheres” (Liu et al., 2017). The name is also abusively employed in this case, as the authors demonstrated that their nanoparticles are composed of crystalline nickel sulfide mixed with amorphous nickel oxide.

## General Conclusion

The oxysulfide family is full of surprises. Most of its members are strictly synthetic because of size and electronegativity differences of oxygen and sulfur. It explains why these compounds were obtained as pure materials and identified relatively late in the history of Chemistry. However, since the 1950's, many compositions have been synthesized, from the simplest ternary compounds to oxysulfide materials containing five or more different atoms.

Among this family, Ln_2_O_2_S must be pointed out. It was the first discovered phase, and represents one of the simplest oxysulfide compositions. It is nearly the only one for which the scientific community proposed various applications especially in the domain of imaging. Consequently, it led to the development of numerous synthetic approaches for Ln_2_O_2_S nanoparticles. Size, morphology, composition, and reactivity of Ln_2_O_2_S nanoparticles are more and more controlled and understood. However, there is still an open door for the study of novel Ln_2_O_2_S nanoparticles. Surprisingly, nanoscaled Ln_2_O_2_S with heavy lanthanides remain hard to obtain. To the best of our knowledge, Tm_2_O_2_S nanoparticles were never prepared. Also, most of the articles focus on the luminescence applications. Yet, in the domain of catalysis for instance, europium, cerium, or ytterbium redox properties have only been poorly explored in such compounds. Lastly, at this point there is no general method to control the size and shape of these nanoparticles on a broad range: only slight adjustments are proposed so far. Such control would enable understanding the influence of the nanoscale on the electric, magnetic, and optical properties. In particular, controlling the nanoparticles thickness could allow to identify the transition between a regime of indirect bandgap (as in the bulk) to the direct one.

Regarding transition metals, their integration in quaternary oxysulfides at bulk scale is successful. Recently, lanthanide-free compositions were obtained. Still, crystalline ternary oxysulfides are very rare, and the characterization of the amorphous products is still incomplete, even if molybdenum or titanium oxysulfide thin films for instance were explored in many studies. One of the challenges is to identify preparation route that lead to crystalline compounds that are easier analyzed by structural techniques. The synthesis of nanoscaled materials with accelerated diffusion processes and possible metastable phases, the developments of characterization techniques and the promising field of applications of transition metals oxysulfides should start an unprecedented area of novel oxysulfide syntheses, identifications and applications.

## Author Contributions

All authors listed have made a substantial, direct and intellectual contribution to the work, and approved it for publication.

### Conflict of Interest

The authors declare that the research was conducted in the absence of any commercial or financial relationships that could be construed as a potential conflict of interest.
